# Murine uterine gland branching is necessary for gland function in implantation

**DOI:** 10.1093/molehr/gaae020

**Published:** 2024-05-24

**Authors:** Katrina Granger, Sarah Fitch, May Shen, Jarrett Lloyd, Aishwarya Bhurke, Jonathan Hancock, Xiaoqin Ye, Ripla Arora

**Affiliations:** Department of Obstetrics, Gynecology and Reproductive Biology, Michigan State University, East Lansing, MI, USA; Institute for Quantitative Health Science and Engineering, Michigan State University, East Lansing, MI, USA; Department of Obstetrics, Gynecology and Reproductive Biology, Michigan State University, East Lansing, MI, USA; Institute for Quantitative Health Science and Engineering, Michigan State University, East Lansing, MI, USA; Department of Obstetrics, Gynecology and Reproductive Biology, Michigan State University, East Lansing, MI, USA; Institute for Quantitative Health Science and Engineering, Michigan State University, East Lansing, MI, USA; Department of Obstetrics, Gynecology and Reproductive Biology, Michigan State University, East Lansing, MI, USA; Institute for Quantitative Health Science and Engineering, Michigan State University, East Lansing, MI, USA; Department of Obstetrics, Gynecology and Reproductive Biology, Michigan State University, East Lansing, MI, USA; Institute for Quantitative Health Science and Engineering, Michigan State University, East Lansing, MI, USA; Department of Physiology and Pharmacology, College of Veterinary Medicine, University of Georgia, Athens, GA, USA; Interdisciplinary Toxicology Program, University of Georgia, Athens, GA, USA; Department of Physiology and Pharmacology, College of Veterinary Medicine, University of Georgia, Athens, GA, USA; Interdisciplinary Toxicology Program, University of Georgia, Athens, GA, USA; Department of Obstetrics, Gynecology and Reproductive Biology, Michigan State University, East Lansing, MI, USA; Institute for Quantitative Health Science and Engineering, Michigan State University, East Lansing, MI, USA

**Keywords:** uterine gland branching, estrogen signaling, embryo implantation, leukemia inhibitory factor, implantation chamber

## Abstract

Uterine glands are branched, tubular structures whose secretions are essential for pregnancy success. It is known that pre-implantation glandular expression of leukemia inhibitory factor (LIF) is crucial for embryo implantation; however, the contribution of uterine gland structure to gland secretions, such as LIF, is not known. Here, we use mice deficient in estrogen receptor 1 (ESR1) signaling to uncover the role of ESR1 signaling in gland branching and the role of a branched structure in LIF secretion and embryo implantation. We observed that deletion of ESR1 in neonatal uterine epithelium, stroma, and muscle using the progesterone receptor *Pgr^Cre^* causes a block in uterine gland development at the gland bud stage. Embryonic epithelial deletion of ESR1 using a Müllerian duct Cre line, *Pax2^Cre^*, displays gland bud elongation but a failure in gland branching. Reduction of ESR1 in adult uterine epithelium using the lactoferrin-Cre (*Ltf^Cre^*) displays normally branched uterine glands. Unbranched glands from *Pax2^Cre^ Esr1^flox/flox^* uteri fail to express glandular pre-implantation *Lif*, preventing implantation chamber formation and embryo alignment along the uterine mesometrial–antimesometrial axis. In contrast, branched glands from *Ltf^Cre^ Esr1^flox/flox^* uteri display reduced expression of ESR1 and glandular *Lif* resulting in delayed implantation chamber formation and embryo–uterine axes alignment but mice deliver a normal number of pups. Finally, pre-pubertal unbranched glands in control mice express *Lif* in the luminal epithelium but fail to express *Lif* in the glandular epithelium, even in the presence of estrogen. These data strongly suggest that branched glands are necessary for pre-implantation glandular *Lif* expression for implantation success. Our study is the first to identify a relationship between the branched structure and secretory function of uterine glands and provides a framework for understanding how uterine gland structure–function contributes to pregnancy success.

## Graphical Abstract

**Graphical Abstract:**
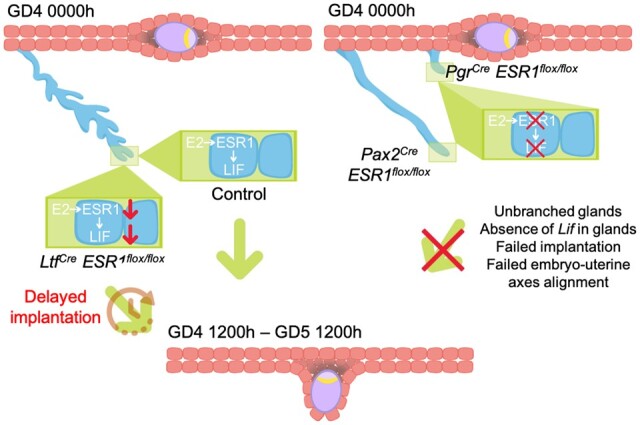
**Control and *Ltf^Cre^ ESR1^flox/flox^* mice with branched glands (blue) display threshold levels of LIF production permitting proper embryo (purple) axis alignment and V-shaped implantation chamber formation (pink cells) allowing embryo development to term.** In contrast, the branchless glands in *Pgr^Cre^ ESR1^flox/flox^* and *Pax2^Cre^ ESR1^flox/flox^* mice compromise LIF secretion, resulting in implantation failure. GD: gestational day; LIF: leukemia inhibitory factor; ESR1: estrogen receptor 1; Ltf: lactoferrin; Pgr: progesterone; Pax2: paired box gene 2.

## Introduction

Uterine glands are key to embryo implantation and pregnancy success. In viviparous mammals in the absence of yolk, uterine glands support the pregnancy and embryo development until the placenta forms (Kelleher *et al.*, 2019). Uterine glands are present in humans, rodents, sheep, pigs, and horses among other studied mammals (Gray *et al.*, 2001). Uteri of neonatal mice are devoid of glands at birth. At postnatal day (P) 5, gland buds protrude off the uterine lumen on the anti-mesometrial and lateral sides of the murine uterus and extend into the surrounding stroma (Vue *et al.*, 2018). Uterine glands are branched in the pubertal non-pregnant and pregnancy stages (Arora *et al.*, 2016). Although uterine glands are categorized as exocrine glands, their mechanisms of branching have not been identified unlike those of other exocrine glands (e.g. salivary glands) (Khan *et al.*, 2022). Uterine glands facilitate the secretions of vital factors, including the key cytokine leukemia inhibitory factor (LIF) that is essential for embryo implantation (Stewart *et al.*, 1992; Chen *et al.*, 2000). Although the relationship between exocrine gland structure and function has been established in the case of meibomian, mammary, and salivary glands (Khan *et al.*, 2022), this relationship in uterine glands is still unknown.

Estrogen (E2) signaling facilitates the growth and development of the uterus as suggested by various estrogen receptor (ESR) deletion models. The ESR has two isoforms, ESR1 and ESR2, which are encoded by separate genes located on different chromosomes. Both receptor isoforms are nuclear receptors and act as ligand-activated transcription factors that can alter target gene transcription (Cui *et al.*, 2013). Female mice with a whole body ESR1 deletion are infertile (Hewitt *et al.*, 2000). These females fail to ovulate and exhibit hypoplastic uteri that fail to display cyclic changes (Dupont *et al.*, 2000). In contrast, females with a whole body ESR2 deletion have sub-fertility with a reduced litter size (Hewitt *et al.*, 2000). Mice with conditional deletion of *Esr1* in different compartments have been generated to highlight the cell-type-specific contributions of ESR1 signaling in the uterus. Deletion of ESR1 in the neonatal epithelium, stroma, and muscle, using progesterone receptor driven *Pgr^Cre^* (where the endogenous *Pgr* gene is replaced by *Cre* recombinase gene) and in embryonic epithelium using *Wnt7a^Cre^* (where the endogenous *Wnt7a* gene is replaced by *Cre* recombinase gene) results in infertility (Winuthayanon *et al.*, 2010; Pawar *et al.*, 2015). Implantation has not been assessed in a *Pgr^Cre^* model and epithelial deletion of ESR1 using *Wnt7a^Cre^* results in protease-mediated embryo death in the oviduct (Winuthayanon *et al.*, 2015). Blastocyst stage embryo transfer studies into pseudopregnant uteri of the latter mice suggest that embryo implantation is not supported in this model (Winuthayanon *et al.*, 2010).

Gland development is variably responsive to ovarian E2 depending on the species. In the neonatal pig, gland development is both ESR1-dependent and sensitive to E2 levels (Tarleton *et al.*, 1999, 2003). Unlike the pig, in sheep, ESR1-inhibition does not impact gland initiation (Carpenter *et al.*, 2003) but reduces the number of glands, and reduces branches and coils in the uterine glands (Carpenter *et al.*, 2003). In neonatal mice, the initiation of gland formation is thought to be ovary-, adrenal-, and steroid-independent (Ogasawara *et al.*, 1983; Bigsby and Cunha, 1985). However, treatment with Genistein (an ESR1 agonist) during the neonatal period prevents gland development and results in implantation failure (Jefferson *et al.*, 2020). Gland structure in *Esr1*-deficient mice has not been assessed.

Mouse mutants with an absence of uterine glands or key glandular secretion of LIF display implantation and decidualization failure (Filant and Spencer, 2013). Examples include: mice-deficient in *Wnt7a* (Dunlap *et al.*, 2011); *Foxa2* (Jeong *et al.*, 2010; Kelleher *et al.*, 2017); progesterone-induced gland knockout (Filant *et al.*, 2012); and *Lif* (Stewart *et al.*, 1992). The luminal epithelium, glandular epithelium, and stromal compartment all possess the ability to express *Lif*, and the coordination of LIF secretion among these compartments presumably happens in accordance with steroid hormone levels during both the mouse estrous cycle and early pregnancy (Rosario and Stewart, 2016). ESR1 signaling is critical for *Lif* induction. Ovulatory E2 coincides with ESR1 and *Lif* expression in the uterus (Shen and Leder, 1992; Song *et al.*, 2000) and, prior to implantation, uterine glands express ESR1 and LIF (Song *et al.*, 2000). A large dose of E2 has the effect of inducing robust *Lif* expression, and this response is absent in ESR1 knockout mice (Hewitt *et al.*, 2002; Winuthayanon *et al.*, 2010). However, the compartment in which *Lif* is expressed (luminal or glandular epithelium) when stimulated by a large dose of E2 remains unknown. *Wnt7a^Cre^* deletion of ESR1 results in a reduction of *Lif* and failed oil-mediated artificial decidualization. Exogenous *Lif* supplementation rescues artificial decidualization in this model (Pawar *et al.*, 2015). This highlights the need for epithelial ESR1 signaling for pre-implantation *Lif* production in early pregnancy.

Studies have demonstrated that there is a link between ESR1 signaling and mammary gland branching such that both the complete abrogation of ESR1 signaling and epithelial-specific deletion of ESR1 result in significantly reduced mammary gland branching and consequently diminished lactational function (Bocchinfuso *et al.*, 2000; Sternlicht *et al.*, 2006; Feng *et al.*, 2007). While the role of ESR1 signaling has been separately studied during uterine development and for pre-implantation *Lif* expression, a connection between gland structure and function is yet to be made. In this study in mice, we determine the relationship between E2 signaling, development of a branched gland structure, and pre-implantation glandular LIF production. We determine that neonatal stromal ESR1 signaling is key to gland elongation and pubertal ESR1 signaling is necessary for gland branching. We also uncovered that unbranched, ESR1-deficient glands fail to express *Lif*, whereas branched glands with much reduced levels of ESR1 can still produce enough LIF to support embryo implantation and pregnancy. Finally, we show that pre-pubertal uteri support *Lif* expression in the luminal epithelium but not in the unbranched glands, suggesting that gland structure (branching) is necessary for gland function (glandular LIF production and implantation).

## Materials and methods

### Animals

*Esr1^flox/flox^* mice (C57Bl6 background) were provided by Dr Pierre Chambon (Gieske *et al.*, 2008). These mice were bred with *Pgr^Cre^* (Soyal *et al.*, 2005) (C57Bl6 background)*, Pax2^Cre^* (Ohyama and Groves, 2004) (mixed background) (where the endogenous *Pax2* gene is replaced by *Cre* recombinase gene), *Wnt7a^Cre^*(Winuthayanon *et al.*, 2010) (C57Bl6 background), or *Ltf^Cre^* (Daikoku *et al.*, 2014) (mixed background) (where the endogenous *Ltf* gene is replaced by *Cre* recombinase gene) mouse lines to generate tissue- and time-specific deletion of ESR1 (Table 1). *Esr1^flox/flox^* females were used as controls. For pregnancy studies, females were mated with fertile CD1 males and the appearance of a vaginal plug was identified as gestational day (GD) 0 1200 h. Adult females >6 weeks were used for *Pgr^Cre^ Esr1^flox/flox^* (hereafter referred to as PER) and *Pax2^Cre^ Esr1^flox/flox^* (hereafter referred to as XER) deletion models. In the *Ltf^Cre^ Esr1^flox/flox^* deletion model (hereafter referred to as LER), because Cre expression comes on at puberty (∼6 weeks), adult females aged 10–12 weeks were used to allow sufficient time for *Esr1* excision. For the pups study, LER virgin females were set up with CD1 males, monitored for pregnancy, and the number of live pups was recorded at birth. *Wnt7a^Cre^ Esr1^flox/flox^* mice (hereafter referred to as WER) (Hewitt *et al.*, 2010) were used for analysis of uterine gland structure to compare with XER mice. These *Esr1^flox/flox^* mice were generated independently by Dr Kenneth Korach (Hewitt *et al.*, 2010); however, the *Esr1^flox/flox^* mice used in our studies have exon 3 of the *Esr1* gene flanked by LoxP sites and result in complete absence of the ESR1 protein. To detect implantation sites, female mice at GD4 1200 h mice were injected i.v. or retroorbitally with blue dye prior to dissection. All animals were maintained and handled according to the Michigan State University Institutional Animal Care and Use Committee guidelines.

**Table 1.** Genotypes of the estrogen receptor 1 murine deletion models and abbreviations used in the study.

Mouse line	Abbreviation	Uterine cell-type in which deletion occurs	Time and tissue of deletion	Ref.
*Pgr^Cre^ ESR1^flox/flox^*	PER	Epithelium, stroma, circular muscle	Neonatal (P5 epithelium, stroma; P21 circular muscle)	Soyal *et al.*, 2005; Madhavan and Arora, 2022
*Pax2^Cre^ ESR1^flox/flox^*	XER	Epithelium	Embryonic (E11.5)	Ohyama and Groves, 2004; Douglas *et al.*, 2012
*Wnt7a^Cre^ ESR1^flox/flox^*	WER	Epithelium	Embryonic (E11.5)	Winuthayanon *et al.*, 2010
*Ltf^Cre^ ESR1^flox/flox^*	LER	Epithelium	Adult (>8.5 weeks)	Daikoku *et al.*, 2014

ESR1, estrogen receptor 1; *Pgr^Cre^ ESR1^flox/flox^*, deletion of ESR1 using progesterone promoter driven CRE expression; *Pax2^Cre^ ESR1^flox/flox^*, deletion of ESR1 using paired box 2 (PAX2) promoter-driven CRE expression; *Wnt7a^Cre^ ESR1^flox/flox^*, deletion of ESR1 using wingless-type MMTV integration site family, member 7A (Wnt7a) promoter-driven CRE expression; *Ltf^Cre^ ESR1^flox/flox^*, deletion of ESR1 using lactoferrin promoter-driven CRE expression; P, postnatal day; E, embryonic day.

### LIF, hormone, and inhibitor treatments

For *Lif* rescue experiments, XER mice were injected i.p. at 1000 and 1800 h on GD3 with either PBS or 10 µg recombinant *Lif* (554006, BioLegend, San Diego, CA, USA) followed by blue dye injection and dissection at GD4 1200 h. Alternatively, one uterine horn of XER mice was injected intraluminally with 1 µg recombinant *Lif* at 1300 h on GD3. The partner horn was left untreated as a control. Mice were then dissected on GD4 1200 h following blue dye injection. For exogenous hormone treatments, 17β-estradiol (E2) (E8875, Sigma-Aldrich, St Louis, MO, USA) and progesterone (P4) (P0130, Sigma-Aldrich) were dissolved in sesame oil and three injection schemes were used. To test *Lif* induction by ovarian hormones in pre-pubertal mice, control P21 mice were injected s.c. with either 100 ng E2 or sesame oil at 1200 on P21 and 0900 h on P22 before dissection at 1200 h on P22. Alternatively, control P21 mice were injected s.c. with 100 ng E2 at 1200 h on P21 and P22. On P23, mice were injected with 1 mg P4 + 6.7 ng E2 at 0900 h and subsequently dissected at 1200 h.

### Cryoembedding, cryosectioning, and immunostaining

For cryoembedding, uterine horns were dissected and fixed in 4% paraformaldehyde overnight at 4°C. The next morning, uteri were washed three times, 5 min each, with PBS and left upright in a solution of 10% sucrose overnight. Uteri were then transferred into 20% and 30% sucrose solutions, sequentially, for 2–3 h each. Finally, uteri were embedded longitudinally in Tissue-Tek OCT (45831, Andwin Scientific, Simi Valley, CA, USA) and stored at −80°C. Tissues were cryosectioned at 7 µm, mounted on glass slides (1255015, Thermo Fisher, Waltham, MA, USA), and stored at −20°C until immunostaining. For ESR1 immunostaining, antigen retrieval was performed by washing slides in PBS before transferring to 1× citrate buffer solution (005000, Thermo Fisher) and boiling in a beaker filled to ¾ in the microwave for 10 min. The slides were then washed three times for 5 min each with PBS, blocked with 2% powdered milk in PBS + 1% Triton X-100 (PBT), and incubated with primary antibodies overnight at 4°C. The next day, slides were washed three times for 5 min each with PBS, stained with secondary antibodies for 1 h, washed with PBS again, and sealed with a coverslip and nail polish.

### Confirmation of Pax2 Cre-lineage

Pax2 lineage was confirmed by breeding the *Pax2^Cre^* mouse line with *Gt(ROSA)26Sor^tm4(ACTB-tdTomato,-EGFP)Luo/J^* also referred to as *ROSA^mT/mG^* (007576, Jackson Labs, Bar Harbor, ME, USA) reporter mice. Uteri from P21 pups and GD3 1200 h pregnant mice were dissected and embedded for cryosectioning. Cryosections were imaged for endogenous membrane green fluorescent protein (GFP) and membrane tomato signal. Some cryosections were additionally stained with primary antibody for CD31 to identify blood vessels (see next section).

### Whole-mount and tissue section immunofluorescence

Whole-mount immunofluorescence was performed as described previously (Arora *et al.*, 2016) Uteri were dissected from mice and fixed in dimethylsulphoxide: methanol (1:4). For immunostaining, uteri were rehydrated in methanol: PBT (1% Triton X-100 in PBS) (1:1) for 15 min, washed in PBT for 15 min and incubated in blocking solution (2% powdered milk in PBT) for 1 h at room temperature. Uteri were incubated with 1:500 concentration of primary antibodies diluted in blocking solution for seven to nine nights at 4°C. They were then washed twice for 15 min each with 1% PBT followed by three washes for 45 min each at room temperature. Uteri were then incubated with secondary antibodies at 4°C for two or three nights, followed by one 15 min and three 45 min washes with 1% PBT and dehydration in 100% methanol for 30 min. Uteri were then bleached overnight at 4°C in a solution of 3% H_2_O_2_ in methanol. Finally, the samples were washed in 100% methanol for 1.5 h and cleared in BABB (1:2, benzyl alcohol: benzyl benzoate) (108006, B6630, Sigma-Aldrich).

### Antibodies

Primary antibodies used include mouse anti-ESR1 (MA5-13191, Thermo Fisher; ab93021, Abcam, Cambridge, UK; 1:200), rat anti-CDH1 (M108, Takara Biosciences, San Jose, CA, USA; 1:500), rabbit anti-FOXA2 (ab108422, Abcam; 1:200), rabbit anti-PTGS2 (ab16701, Abcam; 1:500), rat anti-CD31 (B553370, BD Biosciences, Franklin Lakes, NJ, USA; 1:200), and Biotin-conjugated isolectin B4 (B1205.5, Vector Laboratories, Newark, CA, USA; 1:200). Alexa Fluor-conjugated secondary antibodies: donkey anti-mouse 555 (1:500), goat anti-rat 647 (A21247, Invitrogen, Carsbad, CA, USA; 1:500), goat anti-rat 633 (A21094, Invitrogen; 1:500), and donkey anti-rabbit 555 (A31572, Invitrogen; 1:500). Hoechst (B2261, Sigma-Aldrich; 1:500) was used to stain the nucleus. Streptavidin Dylight 633 (21844, Invitrogen; 1:500) was used as a secondary antibody for isolectin B4.

### *In situ* hybridization

*In situ* hybridization on uterine sections was performed using the RNAscope 2.5 HD Assay-RED kit (322350, ACD Bio, Newark, CA, USA), which incorporates immunofluorescence capabilities. A Mm-*Lif* probe (475841, ACD Bio) was used to detect *Lif* mRNA and immunostaining for FOXA2 was included to label uterine glands. The technique was carried out according to the protocols outlined by ACD Bio (322360-USM, MK 51-149 TN). Primary and secondary antibodies used include rabbit anti-FOXA2 (ab108422, Abcam; 1:200) and Alexa-Fluorophore donkey anti-rabbit 647 (A31573, Invitrogen; 1:500), respectively. Hoechst (B2261, Sigma-Aldrich; 1:500) was used to stain the nucleus.

### Confocal microscopy

Samples with whole tissue immunofluorescence, section immunofluorescence and *in situ* hybridization were all imaged using a Leica TCS SP8 X Confocal Laser Scanning Microscope System (Leica, Wetzlar, Germany) with white-light laser, 10× air objective (used for whole tissue) and 20× water objective (used for sections). The entire length and thickness of the uterine horn was imaged using the tile scan function with *z* stacks 7 μm apart. For sections, *z* stacks 1.5 μm apart were used. Images were merged using Leica software LASX version 3.5.5 (Leica).

### 3D Reconstruction and image analysis

Image analysis was performed using commercial software Imaris v9.2.1 (Bitplane; Oxford Instruments, Abingdon, UK). The confocal image (.LIF) files were imported into the Surpass mode of Imaris.

#### Gland visualization

The Surface function of Imaris was used to reconstruct 3D gland surfaces based on the FOXA2 fluorescent signal. The Imaris Vantage function was used to isolate individual glands into a comprehensive gallery for visualization.

#### Quantitative analysis of gland length and branch numbers

The images of uterine horns were imported into Imaris v9.2.1 (Bitplane) with MATLAB (XT) module (MathWorks, Natick, MA, USA). The surface function in Imaris was used to create 3D renderings of uterine glands from the fluorescent staining by background subtraction with the diameter of the largest sphere set to 30. With files of uterine horns stained only with cytokeratin 8, glands were isolated manually from the surface by using the scissors tool to cut them away from the lumen. For gland length, the Bounding Box OOC function in Imaris was used that determines the shortest straight-line distance from the point where the gland is connected to the uterine lumen to the furthest tip. Masks of the gland surfaces were made to get a channel with uniform gland signal throughout. These channels were then imported to FIJI (ImageJ). Thresholding was used to binarize the isolated image and the ‘Fill Holes’ function was used to fill in any gaps to ensure a more accurate skeleton. This 3D image was then saved as a TIFF file and imported into MATLAB. The ‘imread’ function was used so MATLAB could read the data and a new variable was defined using the command ‘uint8(Skeleton3D(imbinarize(stack)))*200;’. This command defines the file as a binary image, calculates the 3D skeleton of a binary volume using a thinning algorithm, and increases the intensity of the signal throughout. The ‘saveastiff’ function was then used to save this revised file as a TIFF file, which was then added to the original Imaris file as a new channel. Filaments were created manually using that channel. The number of dendrite branch points was exported from Imaris in a Microsoft Excel (Microsoft, Redmond, WA, USA) file for statistical analysis (Khan *et al.*, 2024).

#### Determination of embryo axis orientation

Embryo axis orientation was determined by identifying embryos via the Hoechst signal and using the Measurement Points module in Imaris. The embryo axis can be determined by identifying the inner cell mass (ICM) or the embryonic pole of the blastocyst and the mural trophectoderm (abembryonic pole). Proper embryo alignment is characterized by the embryonic pole facing the mesometrial side of the uterus and the abembryonic pole facing the anti-mesometrial side of the uterus. The embryo at an implantation site on GD4 was visualized using an optical XY Orthogonal Slicer or Oblique Slicer. An XZ Orthogonal Slicer was used to define the mesometrial–anti-mesometrial (M-AM) axis and was placed at the abembryonic pole of the embryo. Using the Measurement Points module, the first point was placed on the ICM on the M-AM plane. The second point was placed on the intersection of the M-AM plane and the abembryonic pole on the XY plane. The third point was placed on the intersection of the M-AM and XY planes. The value of the angle was obtained using the Statistics function of Imaris.

#### Quantifying the Lif signal

For quantifying the *Lif* signal from *in situ* hybridization on cryosections, the Imaris Surface function was used to construct volumetric surfaces of gland nuclei, and *Lif* signal. For quantitation of *Lif* volume per gland volume, volumetric glandular surface of the *z*-stack of a single cross section of uterus was constructed according to the FOXA2 signal. Then, the Imaris masking function was used to create a separate channel of *Lif* signal underneath the previously constructed gland surface volume. Based on this *Lif* signal channel, volumetric *Lif* surface was created. The Statistics function of Imaris was utilized to determine gland and *Lif* surface volumes, and Microsoft Excel was implemented to calculate *Lif* volume per gland volume.

### Statistical analysis

To compare the numbers of implantation sites, gland length measurements of P21 treatment mice, embryo axis orientation, and *Lif* volume per gland volume, the Kruskal–Wallis test with Dunn’s multiple comparisons was conducted. For analysis of the number of implantation sites/pups, gland length measurements of GD4 1200 h and P21 mice, and *Lif* volume per lumen volume, the Mann–Whitney test was applied. To assess whether the proportions were comparable in ESR1-stained sections and the number of glands with various branches, a two-proportion *Z*-test was used. Statistical analyses were performed using GraphPad Prism (Dotmatics; GraphPad, La Jolla, CA, USA) with advanced statistical analyses conducted using R Statistical Software (R Core Team, 2014). A *P*-value <0.05 was considered significant, indicating differences between comparisons.

## Results

### Generation of tissue-specific ESR1 deletion mice

To assess the role of ESR1 in uterine gland structure, we generated mice with uterine-specific deletion of ESR1 in a time and compartment-specific manner (Table 1). *ESR1^flox/flox^* mice bred with a *Pgr^Cre^* mouse line (*Pgr^Cre^ ESR1^flox/flox^*, hereafter referred to as PER) were generated to determine the role of neonatal epithelial, stromal, and muscle-derived ESR1 (Madhavan and Arora, 2022). To determine the role of epithelial ESR1 in gland structure, we used a *Pax2^Cre^* mouse line to generate mice where ESR1 was deleted in the embryonic Müllerian duct epithelium (*Pax2^Cre^ ESR1^flox/flox^*, hereafter referred to as XER). To determine the role of epithelial ESR1 in gland structure in the post-pubertal mouse, a *Ltf^Cre^* mouse line was used (*Ltf^Cre^ ESR1^flox/flox^*, hereafter referred to as LER).

**Figure 1.**
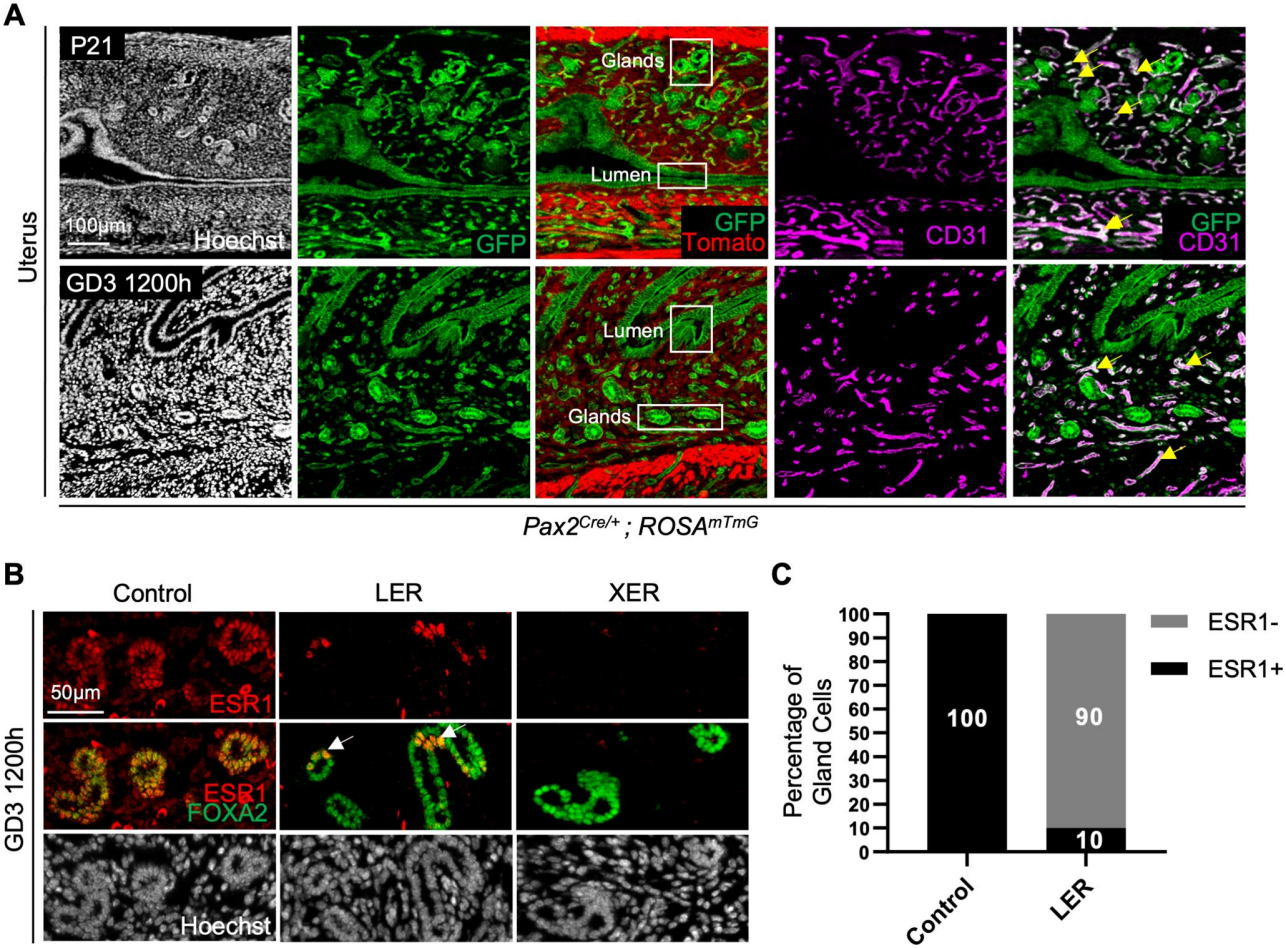
**Uterine compartment-specific excision of estrogen receptor 1 in mice.** (**A**) *Pax2^Cre/+^*; *ROSA^mTmG^* mouse uterine section at P21 and GD3 1200 h with Hoechst (nuclei, white), GFP (green), Tomato (red), and CD31 (endothelial cell marker, magenta) identifying Pax2 lineage cells (green) in the luminal epithelium, glandular epithelium, and vasculature. Scale bar, 100 μm; yellow arrows indicate colocalization of GFP and CD31. (**B**) Uterine sections from control (6–8 weeks old), LER (12–14 weeks old), and XER (6–8 weeks old) mice identifying ESR1 (red) and FOXA2 (gland marker, green). XER mice show complete ablation of ESR1 while LER mice have few gland cells that maintain ESR1 expression (white arrowheads). Scale bar, 50 μm. n = 3–5 mice per genotype. (**C**) Quantification of the percentage of ESR1+ gland cells in LER glandular epithelium compared to controls. Controls (n = 4 mice, 9 sections, 2848 gland cells); LERs (n = 5 mice, 17 sections, 3250 gland cells). Two-proportion *Z*-test determined that the differences in proportions of gland cells expressing ESR1 between control and LERs was statistically significant. Pax2, paired box gene 2; P, postnatal day; GD, gestational day; GFP, green fluorescent protein; CD31, cluster of differentiation 31; ESR1, estrogen receptor 1; FOXA2, forkhead box A2; LER, *Ltf^Cre^ ESR1^flox/flox^* (adult uterine epithelial deletion of ESR1 using lactoferrin promoter driven CRE expression); XER, *Pax2^Cre^ ESR1^flox/flox^* (embryonic uterine epithelial deletion of ESR1 using paired box 2 (PAX2) promoter driven CRE expression).

Since this is the first application for *Pax2^Cre^* in the uterine epithelium, we discerned the lineage of *Cre* expressing cells using the *ROSA^mT/mG^* (Muzumdar *et al.*, 2007) reporter line. We observed that Pax2 lineage labeled cells contribute to both the luminal and glandular epithelium of the uterus but are absent from the muscle and stroma on P21 and gestational day (GD) 3 1200 h (Fig. 1A). We also observed that the oviduct displays partial expression of the lineage label at P21 (Supplementary Fig. S1). In addition to the luminal and glandular epithelium, the CD31+ vascular compartment also expresses the lineage label (GFP) (Fig. 1A). Immunostaining with ESR1 and the vascular marker isolectin suggested no expression of ESR1 in endothelial cells at P21, P28, proestrus stage, and GD3 1200 h (Supplementary Fig. S2).

To confirm ESR1 deletion in the glandular epithelium of LER and XER mice, immunological staining with ESR1 and glandular marker FOXA2 was performed (Fig. 1B). While XER mice displayed no expression of ESR1, 10% of LER glandular epithelial cells maintained ESR1 expression at GD3 1200 h (Fig. 1C), suggesting the *Ltf^Cre^* deletion of ESR1 is not 100% complete. Thus, LER mice used for further analysis below are not completely ESR1-deficient but have reduced levels of ESR1.

### Embryonic epithelial ESR1 deletion compromises gland branching

**Figure 2.**
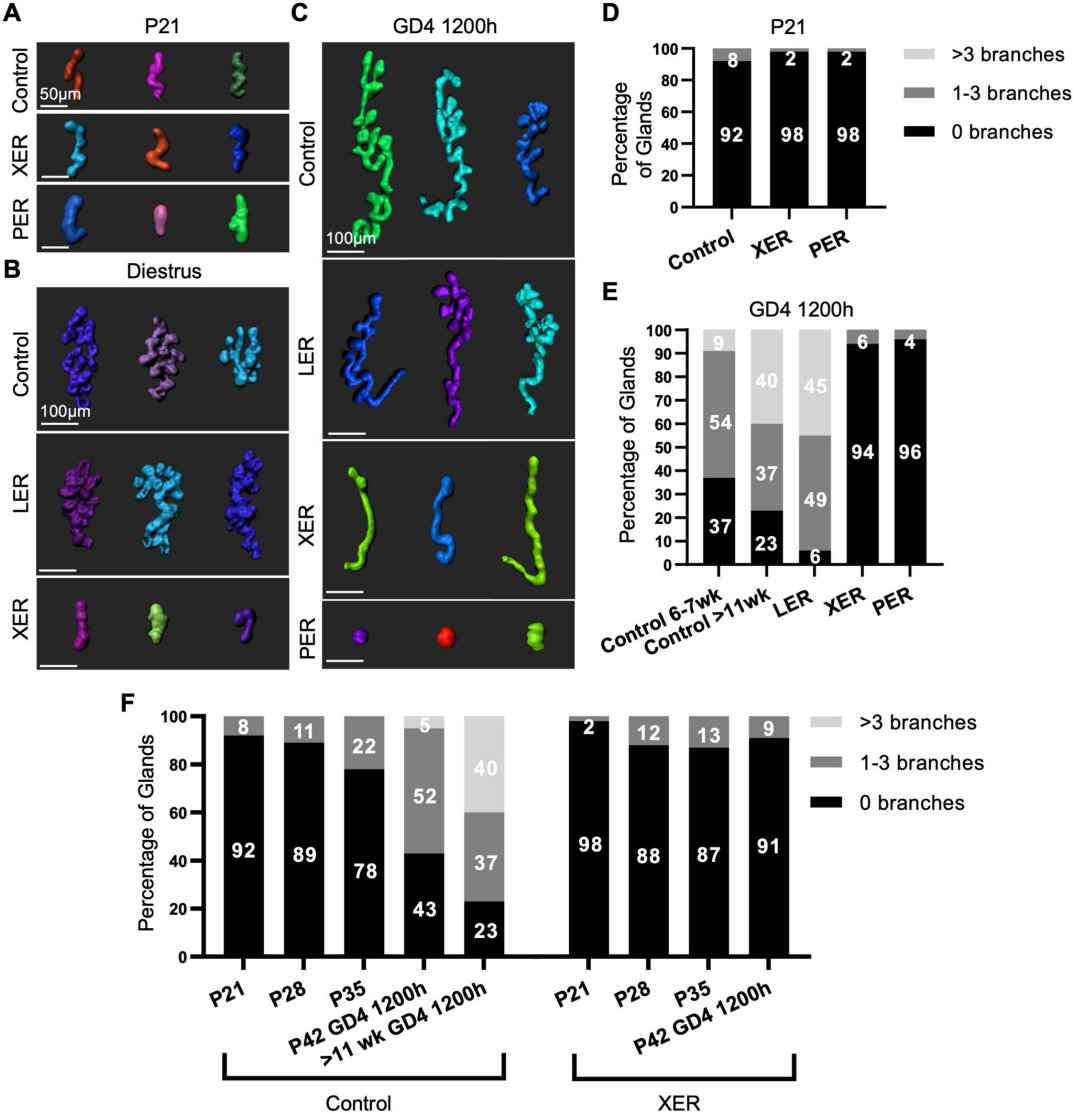
**Structural aberrations in estrogen receptor 1-deficient uterine glands during development and pregnancy in mice**. (**A**–**C**) Representative 3D reconstructions of glands at P21 (A), diestrus (B), and GD4 1200 h (C). Scale bar: 50 μm in A and 100 μm in B and C. n = 3 mice per genotype. At P21, XER and PER glands show comparable branching patterns to controls, but at GD4 1200 h XER and PER glands are unbranched while control and LER glands are branched. (**D** and **E**) Quantitative analysis of the percentage of glands with 0, 1–3, and >3 branches at P21 (D) and GD4 1200 h (E). n = 3 mice per genotype. 75–400 glands analyzed per mouse. (E) Analysis of control glands is separated by age to highlight age-dependent branching effects. Ages of LER mice are >11 weeks and ages of XER and PER mice are between 7 and 16 weeks. Colors in (D–F) correspond to the number of branches. Two-proportion *Z*-test was used to determine statistical differences between groups for the percentage of branched glands. At GD4 1200 h, gland branches between control and XER glands, and control and PER glands are statistically significantly different but there is no significant difference between control and LER or between XER and PER glands. (**F**) Quantitative analysis of the percentage of glands with 0, 1–3, and >3 branches at P21, P28, P35, and P42 GD4 1200 h in control and XER mice. Control mice >11 weeks at GD4 1200 h is also included for visualization of branching progression with age. Control sample sizes: (P21) n = 5, (P28) n = 3, (P35) n = 3, (P42 GD4 1200 h) n = 6, (>11week GD4 1200 h) n = 3. XER sample sizes: (P21) n = 2, (P28) n = 3, (P35) n = 4, (P42 GD4 1200 h) n = 2. 100–850 glands analyzed per mouse. P, postnatal day; GD, gestational day; ESR1, estrogen receptor 1; XER, *Pax2^Cre^ ESR1^flox/flox^* (embryonic uterine epithelial deletion of ESR1 using paired box 2 (Pax2) promoter-driven CRE expression); PER, *Pgr^Cre^ ESR1^flox/flox^* (neonatal uterine epithelial, stromal, and circular muscle deletion of ESR1 using progesterone promoter-driven CRE expression); LER, *Ltf^Cre^ ESR1^flox/flox^* (adult uterine epithelial deletion of ESR1 using lactoferrin promoter driven CRE expression).

Uterine glands in mice develop in a sequential manner from a bud phase to an elongated phase in the pre-pubertal period, eventually becoming branched in adulthood. We sought to analyze gland structure in ESR1 deletion mice at different stages (Fig. 2, Table 2). At P21, a stage when the majority of glands are unbranched (Vue *et al.*, 2018), PER and XER mice display gland buds that are comparable to controls (Fig. 2A). Diestrus, a pubertal, non-pregnant stage, displays branched glands in control uteri (Arora *et al.*, 2016). While glands of LER mice (reduced levels of ESR1) exhibited branching similar to controls, glands of XER mice (no ESR1) displayed unbranched glands (Fig. 2B). To assess gland structure during pregnancy, we analyzed GD4 1200 h uterine glands (Fig. 2C, Supplementary Fig. S3). Control and LER pregnant uteri at GD4 1200 h displayed glands that were branched, whereas XER glands continued to be unbranched but elongated, and PER glands appeared as gland buds (Fig. 2C, Supplementary Fig. S3). Using an image segmentation algorithm (Khan *et al.*, 2024), we quantified gland branching to support our qualitative analysis. At P21, over 90% of glands in control, PER, and XER mice are unbranched, and only 8% of control glands display 1–3 branches. Neither control nor PER or XER glands display any glands with >3 branches at this stage (Fig. 2D). At GD4 1200 h, even within controls, we observed an age-dependent effect on gland branching. For controls at 6–7 weeks, 9% of glands displayed >3 branches, and this number increased to 40% in controls aged >11 weeks (Fig. 2E). LER mice are analyzed at >11 weeks (to allow sufficient CRE-mediated ESR1 excision) and 45% of LER glands display >3 branches at GD4 1200 h. Glands in XER and PER mice failed to display >3 branches irrespective of age evaluated (Fig. 2E, age range 7–16 weeks). In addition to gland branching, we also observed that while gland length was similar in controls, PERs, and XERs at P21, gland length was greatly reduced in GD4 1200 h PERs and XERs (Supplementary Fig. S4). However, LERs displayed a gland length similar to controls at GD4 1200 h (Supplementary Fig. S4).

**Table 2.** Summary of gland branching patterns and functional outcomes in estrogen receptor 1 deletion mice.

		Gland structure characteristics			Functional outcome		
Mouse line	Abbrev.	P21	Diestrus	**GD4 1200** **h**	Implantation	Embryo alignment	*Lif* expression
Control		Unbranched, elongated	Branched	Branched	Normal	Normal	Normal
*Ltf^Cre^ ESR1^flox/flox^*	LER	NA*	Branched	Branched	Delayed	GD4 1200 h: Misaligned	Reduced, threshold level met
						GD5 1200 h: Aligned	
*Pax2^Cre^ ESR1^flox/flox^*	XER	Unbranched, elongated	Unbranched, elongated	Unbranched, elongated	Failed	GD4 1200 h: Misaligned	Absent, threshold level not met
						GD4 1800 h: Embryos have died	
*Pgr^Cre^ ESR1^flox/flox^*	PER	Unbranched, buds	ND	Unbranched, buds	NA**	NA**	NA**

ESR1, estrogen receptor 1; LER, *Ltf^Cre^ ESR1^flox/flox^* (adult uterine epithelial deletion of ESR1 using lactoferrin promoter driven CRE expression); XER, *Pax2^Cre^ ESR1^flox/flox^* (embryonic uterine epithelial deletion of ESR1 using paired box 2 (Pax2) promoter-driven CRE expression); PER, *Pgr^Cre^ ESR1^flox/flox^* (neonatal uterine epithelial, stromal and circular muscle deletion of ESR1 using progesterone promoter driven CRE expression); P, postnatal day; GD, gestational day; ND, not determined; NA, not applicable.

* LERs are only evaluated at >10 weeks.

** PERs do not have embryos so they were not analyzed during pregnancy.

Since XER glands display complete loss of ESR1 (Fig. 1B), we performed an age-dependent comparison for controls and XERs to observe the dynamics of gland branching. At P28 (4 weeks of age, Fig. 2F) control and XER glands again display largely unbranched glands. At P35 (5 weeks of age, Fig. 2F), glands in controls begin to display an increased number of glands with 1–3 branches (22%) when compared to the XER glands (13%). For cycling mice, for consistency of ovarian hormone levels, we compared similar aged mice for gland branching at GD4 1200 h. We observed that at P42 (6 weeks of age, GD4 1200 h, Fig. 2F), control glands continue to display increased branch numbers when compared to XER mice. Within control mice, gland branching at GD4 1200 h for 6-week old mice and mice >11 weeks was significantly different (Fig. 2F and Supplementary Table S1) suggesting glands continue to branch with age.

To determine if gland branching defects in XERs were caused by ESR1 deletion in the embryonic epithelium and not the vasculature, we employed another Müllerian duct Cre *Wnt7a^Cre^* to delete ESR1 in the embryonic epithelium (Table 1). Similar to XERs, *Wnt7a^Cre^ ESR1^flox/flox^* mice (hereafter referred to as WERs) displayed highly reduced gland branching (Supplementary Fig. S5A and B). In WERs, 61% of glands displayed no branching, 34% of glands displayed 1–3 branches and a small proportion displayed >3 branches (5% glands) (Supplementary Fig. S5C). In agreement with previous reports, we observed FOXA2 expression in the luminal epithelium of WERs at GD3 1200 h (Supplementary Fig. S5A) and no embryos were observed in the uterine lumen. These results suggest that while both WNT7A and PAX2 are expressed in the embryonic Müllerian duct epithelium (Carroll *et al.*, 2005), there are differences in *Wnt7a* and *Pax2* promoter-driven CRE expression that result in differential excision of *Esr1* and resulting uterine gland branching. Despite this, the extent of branching in WER uterine glands was greatly reduced compared to controls (Supplementary Fig. S5C). Altogether, these observations suggest that ESR1 plays an important role in determining uterine gland elongation and branching during uterine gland development.

### Branchless glands fail to support pregnancy

**Figure 3.**
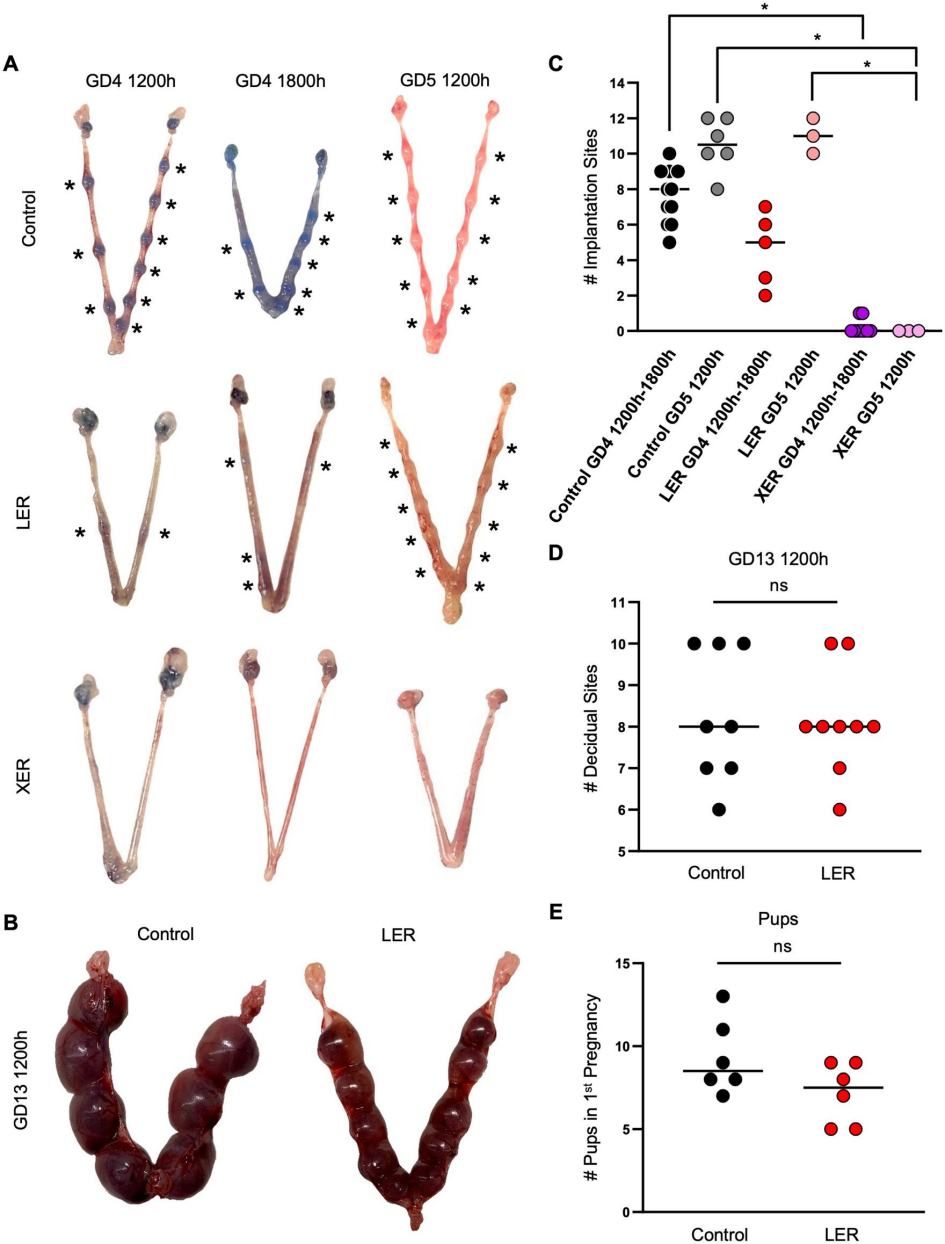
**Implantation and pregnancy phenotypes of mice with uterine epithelial estrogen receptor 1 deletion.** (**A**) Dissected uteri from control, LER, and XER mice at GD4 1200 h, GD4 1800 h, and GD5 1200 h. Uteri at GD4 1200 h and GD4 1800 h were injected with blue dye prior to dissection for visualization of implantation sites. Asterisks indicate implantation/decidual sites. XER mice exhibit failed implantation while LER mice exhibit delayed implantation. (**B**) Dissected uteri from control and LER mice at GD13 1200 h showing that LER mice display pregnancy progression comparable to controls. (**C** and **D**) Quantification of the number of implantation sites at GD4 1200–1800 h and GD5 1200 h in control, LER, and XER mice (C) and at GD13 1200 h in control and LER mice (D). Each dot represents one uterus analyzed. Median values shown. Data analyzed using Mann–Whitney test. (**E**) Quantification of number of live pups born in first pregnancies of control and LER mice. Each dot represents one uterus analyzed. Median values shown. Data analyzed using Mann–Whitney test. (*) = *P* *<* *0.05*. (ns) = *P* > 0.05. ESR1, estrogen receptor 1; GD, gestational day; LER, *Ltf^Cre^ ESR1^flox/flox^* (adult uterine epithelial deletion of ESR1 using lactoferrin promoter driven CRE expression); XER, *Pax2^Cre^ ESR1^flox/flox^* (embryonic uterine epithelial deletion of ESR1 using paired box 2 (PAX2) promoter driven CRE expression).

To determine whether gland branching defects result in functional deficits, we evaluated ESR1-deficient mice at GD3 1200 h, GD4 1200 h, GD4 1800 h, and GD5 1200 h. Since WERs show a failure of blastocyst development in the oviduct, we wanted to first confirm that embryos did indeed enter the uterine lumen in XERs. At GD3 1200 h, when embryos are expected in the uterine horn (Carroll *et al.*, 2005), we observed that one-third of the XER mice displayed embryos in the oviduct only, one-third of the mice displayed embryos in both the oviduct and the uterus, and one-third of the mice displayed embryos in the uterus alone (Table 3). When evaluated a day later at GD4 1200 h, 4/7 XER mice displayed embryos in their uterus and the average number of embryos per mouse was lower than controls (Table 3). At GD4 1200 h, implantation sites were clearly observed in control mice using the blue dye reaction (Psychoyos, 1961) (Fig. 3A). XER mice failed to display any implantation at all, and LER mice displayed faint implantation sites indicating delayed implantation (Lufkin *et al.*, 2023) (Fig. 3A–C). When analyzed a day later for decidual sites at GD5 1200 h, control and LER mice displayed visible deciduae whereas XER mice failed to show decidualization (Fig. 3A and C). Since LERs exhibited delayed implantation, we assessed both embryo development at mid-gestation and the ability of LER females to deliver pups. LER females had apparently normal embryos at GD13 1200 h (Fig. 3B and D) and displayed a normal number of pups at birth (Fig. 3E). Thus, despite the loss of ESR1 in 90% of their glandular epithelium in the pre-implantation stage, LER mice can carry pregnancy to term.

**Table 3.** Embryo location in mice with embryonic epithelial deletion of estrogen receptor 1.

	GD3 1200 h			GD4 1200 h			
	No. of Mice with embryos in oviduct	No. of mice with embryos in oviduct + uterus	No. of mice with embryos in uterus	No. of mice with embryos	No. of mice with no embryos	Total no. of embryos	No. of embryos/ no. of mice with embryos
Control	1/12	0/12	11/12	5	0	36	7.2
XER	2/6	2/6	2/6	4	3	17	4.25

Table indicates location of embryos in control and XER mice at GD3 1200 h. Number of control and XER mice with and without embryos at GD4 1200 h is also indicated along with total number of embryos observed and the average number of embryos with respect to mice where embryos are present in the uterus.

GD, gestational day; XER, *Pax2^Cre^ ESR1^flox/flox^* (embryonic uterine epithelial deletion of ESR1 using paired box 2 (Pax2) promoter-driven CRE expression.

### Mice with branchless glands fail to form an implantation chamber and display aberrant embryo-uterine axes alignment

**Figure 4.**
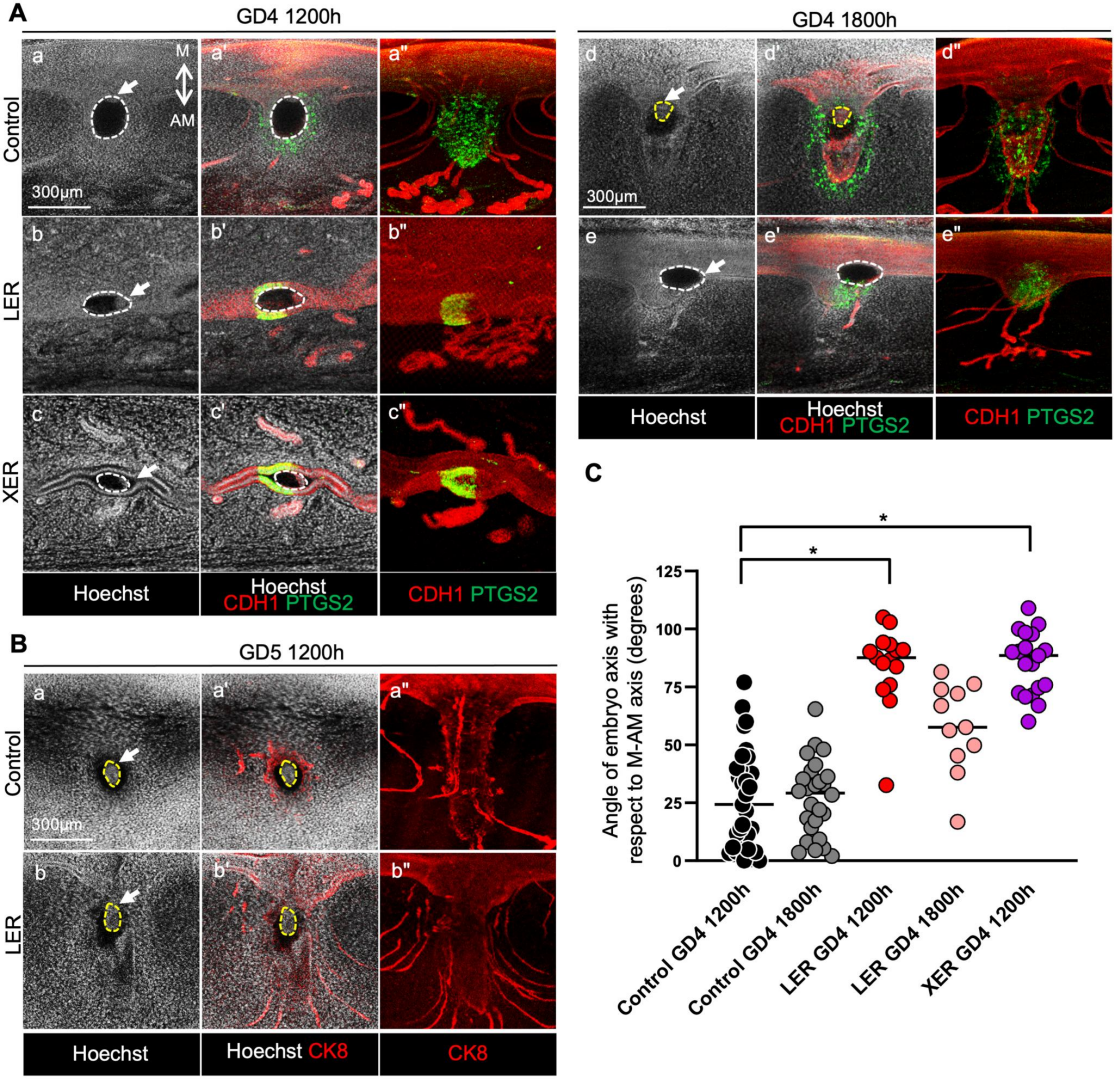
**Embryo-uterine axes alignment phenotypes in mice with uterine epithelial estrogen receptor 1 deletion.** (**A** and **B**) Immunofluorescent images of embryos at implantation sites in control, LER, and XER mice at GD4 1200 h, GD4 1800 h, and GD5 1200 h with staining for (A) Hoechst (nuclei, white), E-Cadherin (epithelium, red), and PTGS2 (green) or (B) Hoechst (nuclei, white) and Cytokeratin 8 (epithelium, red). Cross-sections were obtained from whole tissue confocal imaging. (″) column images represent pseudo-3D cross-sections that are ∼150–300 μm in thickness. All other images are 7 μm in thickness. Mesometrial-antimesometrial (M-AM) axis is indicated (a), and white arrows point to the inner cell mass of embryos. White dotted lines represent the boundary of an embryo. Yellow dotted lines indicate the boundary of the epiblast. Scale bar, 300 μm. (n = 3) mice per genotype per stage. (**C**) Angle measurements of embryo-uterine axis in control, LER, and XER mice at GD4 1200 h and GD4 1800 h. Angle measurements taken with respect to the M-AM axis. Each dot represents one embryo. Median values shown. Data analyzed using Kruskal–Wallis test with Dunn’s multiple comparisons. (*) = *P* *<* 0.05. GD, gestational day; ESR1, estrogen receptor 1; LER, *Ltf^Cre^ ESR1^flox/flox^* (adult uterine epithelial deletion of ESR1 using lactoferrin promoter-driven CRE expression); XER, *Pax2^Cre^ ESR1^flox/flox^* (embryonic uterine epithelial deletion of ESR1 using paired box 2 (PAX2) promoter-driven CRE expression).

Previous research from our laboratory has demonstrated that the formation of a V-shaped embryo implantation chamber is necessary for the alignment of the embryonic and the uterine axes, such that the ICM of the blastocyst faces the mesometrial pole of the uterus (Madhavan *et al.*, 2022). This event coincides with a shift of PTGS2 (prostaglandin synthase 2) expression from the luminal epithelium to the stroma underlying the implantation chamber (Madhavan *et al.*, 2022). When we evaluated ESR1-deficient uteri at GD4 1200 h, we observed that both XERs and LERs showed a failure of V-shaped implantation chamber formation; embryo–uterine axes alignment; and PTGS2 expression in the stroma under the embryo attachment site (Table 2, Fig. 4A a–c″). Control embryos displayed an array of axis angles with a median value of 24.30^°^ (Fig. 4C), while embryos of LER and XER mice were misaligned with the ICM oriented in a perpendicular direction (median angle LER 87.70^°^ and XER 88.6^°^) with respect to the M-AM axis (Fig. 4C). However, at GD4 1800 h, LERs began to display a small V-shaped chamber, improved alignment of the embryo–uterine axis (median angle 57.70^°^), and PTGS2 expression in the stroma underlying the chamber (Fig. 4A d–e″ and C). Further, by GD5 1200 h, both control and LER uteri displayed comparable embryo development commensurate with the epiblast stage (Fig. 4B). We failed to observe live embryos in XER mice at GD4 1800 h (one misaligned embryo was observed in 1/10 uterine horns from five mice analyzed), suggesting that in the absence of branched glands and ESR1, attachment fails at GD4 1200 h and embryos fail to survive beyond this stage (Table 2).

### ESR1-deficient unbranched glands fail to express preimplantation *Lif*

**Figure 5.**
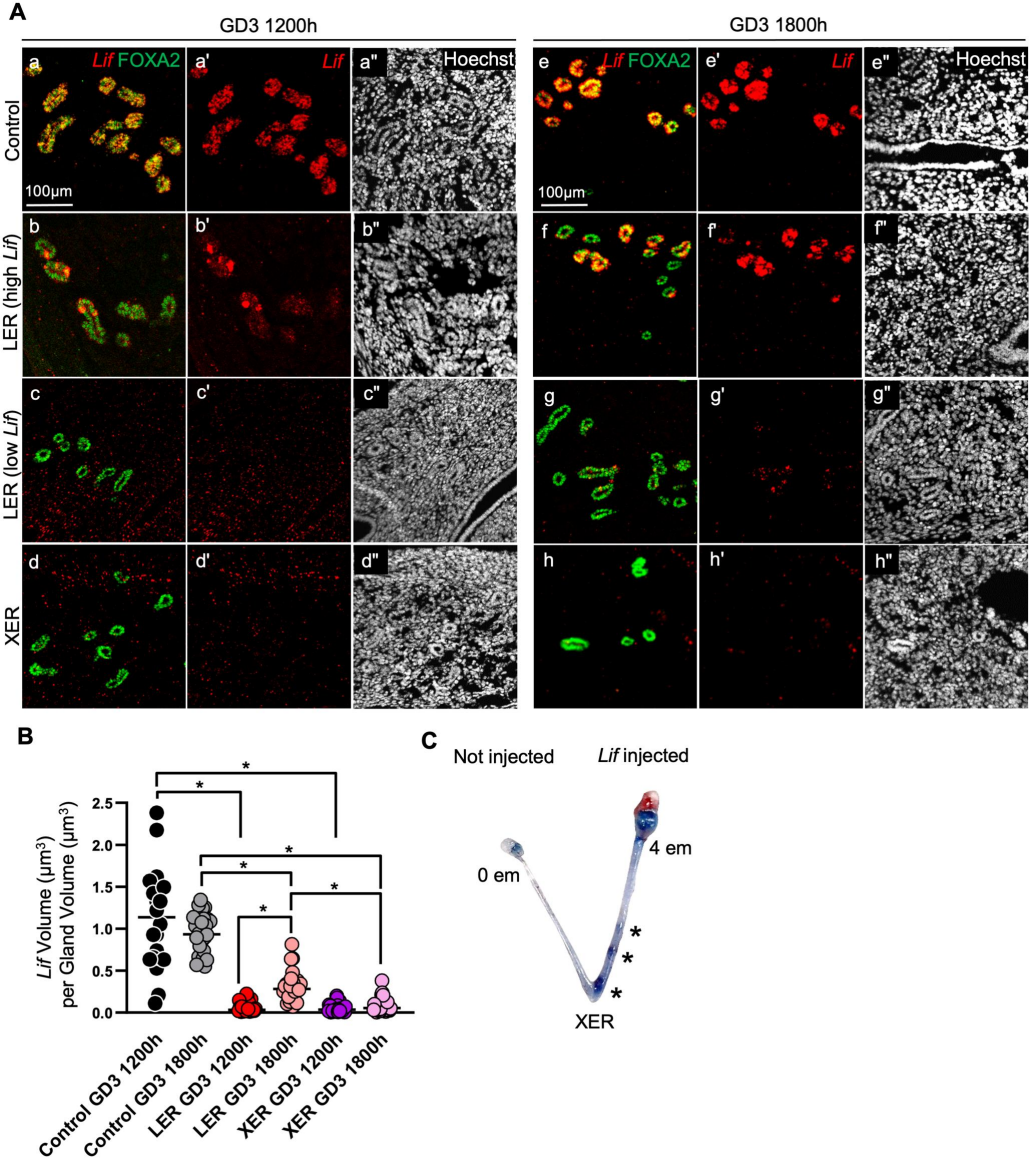
**Epithelial estrogen receptor 1 deletion mice display defects in leukemia inhibitory factor expression.** (**A**) Uterine sections of control, LER, and XER mice at GD3 1200 h and GD3 1800 h with staining for Hoechst (nuclei, white), FOXA2 (gland marker, green), and *Lif* mRNA (red). *Lif* expression in LER glands is high in a subset of cells (b–b″, g–g″) and lower in others (c–c″, g–g″). s*Lif* expression increases from GD3 1200 h to GD3 1800 h in LER glands while XER glands express undetectable levels of *Lif* at both time points. Scale bar, 100 μm. (**B**) Quantitative analysis of the amount of *Lif* per gland volume (normalized units) in control, XER, and LER mice at GD3 1200 h and GD3 1800 h. n = 9 regions each from three mice per genotype per stage were analyzed. Data in (B) analyzed using Kruskal–Wallis test with Dunn’s multiple comparisons. (*) = *P* < 0.05. (**C**) Uterine horn of XER mouse intraluminally injected with 1 μg recombinant *Lif* into the right horn on GD3 1200 h and dissected on GD4 1200 h following blue dye injection. Asterisks indicate implantation rescue sites, and the total number of embryos found in each horn is shown. ESR1, estrogen receptor 1; LER, *Ltf^Cre^ ESR1^flox/flox^* (adult uterine epithelial deletion of ESR1 using lactoferrin promoter driven CRE expression); XER, *Pax2^Cre^ ESR1^flox/flox^* (embryonic uterine epithelial deletion of ESR1 using paired box 2 (PAX2) promoter-driven CRE expression); FOXA2: forkhead box A2; LIF, leukemia inhibitory factor; GD, gestational day; em, embryo.

Since ESR1-deficient mouse models have shown reduced LIF expression (Pawar *et al.*, 2015) we determined *Lif* mRNA expression using *in situ* hybridization. First, we observed that in control mice at GD0 1200 h, *Lif* is highly expressed in the luminal compartment but not in the glandular compartment (Supplementary Fig. S6). At pre-implantation stage GD3 1200 h in controls, strong *Lif* expression was observed colocalizing with FOXA2-expressing uterine gland cells (Fig. 5A, Supplementary Fig. S6) and *Lif* expression was not observed in the luminal compartment. XER glands failed to express *Lif* while, surprisingly, a small amount of *Lif* was observed in LER glands (Fig. 5A and B). Since we observed delayed implantation in LERs, we tested for *Lif* expression a few hours later at GD3 1800 h. We discovered that LER glands showed an increase in *Lif* expression compared to GD3 1200 h (Fig. 5A and B). We quantified total *Lif* volume normalized for gland volume using image segmentation protocols. Control glands express a uniformly high volume of *Lif* normalized for gland volume at GD3 1200 h and GD3 1800 h while LER and XER mice exhibited a much lower volume of *Lif* at GD3 1200 h. We observed a slight but significant increase in the amount of *Lif* volume in LER mice at GD3 1800 h (Fig. 5B). Visually, we observed that although LER gland cells do not uniformly express *Lif*, a few gland cells express exaggerated amounts of *Lif*, sufficient to support implantation (Table 2). Since 10% of LER gland cells continue to make ESR1 (Fig. 1B), *Lif* expression could be occurring in residual ESR1-expressing gland cells or because LERs display branched glands and branching is critical for gland function and *Lif* synthesis.

### *Lif* supplementation partially rescues implantation in ESR-1 deficient uteri

We observed almost no *Lif* expression in XERs, therefore we assessed whether recombinant Lif administration at GD3 could rescue implantation in XER mice at GD4 1200 h (Table 4). For XER mouse uteri that did contain blastocysts, using both i.p. and intraluminal routes of LIF administration (Jeong *et al.*, 2010; Kelleher *et al.*, 2017), we achieved 75% and 67% implantation rescue, respectively (Fig. 5C, Supplementary Fig. S7). Altogether, this demonstrates that the critical difference between XER mice with unbranched glands and LER mice with branched glands is the presence of a threshold amount of LIF in LER mice.

**Table 4.** Implantation rescue in mice with embryonic epithelial deletion of estrogen receptor 1 supplemented with leukemia inhibitory factor.

	Intraluminal		Intraperitoneal	
	No. of mice	No. of embryos/IS	No. of mice	No. of embryos/IS
No. of XER mice injected	7	—	10	—
No. of XER mice with embryos	4	12	6	27
No. of XER mice with IS	3	7	4	11
% Rescue	75% (3/4)	58%	67% (4/6)	41%

Table indicates the total number of XER mice injected either intraluminally (1 μg) or intraperitoneally (10 μg) with leukemia inhibitory factor, the number of mice where embryos were observed in the uterus, the number of mice with implantation sites, and the percentage of rescue of mice with implantation sites compared to mice with embryos. The total number of embryos observed in the uterus and the total number of implanted embryos along with the corresponding percentage rescue is also indicated.

ESR1, estrogen receptor 1; IS, implantation site; XER, *Pax2^Cre^ ESR1^flox/flox^* (embryonic uterine epithelial deletion of ESR1 using paired box 2 (Pax2) promoter-driven CRE expression).

### Unbranched glands with E2–ESR1 signaling fail to express *Lif*

**Figure 6.**
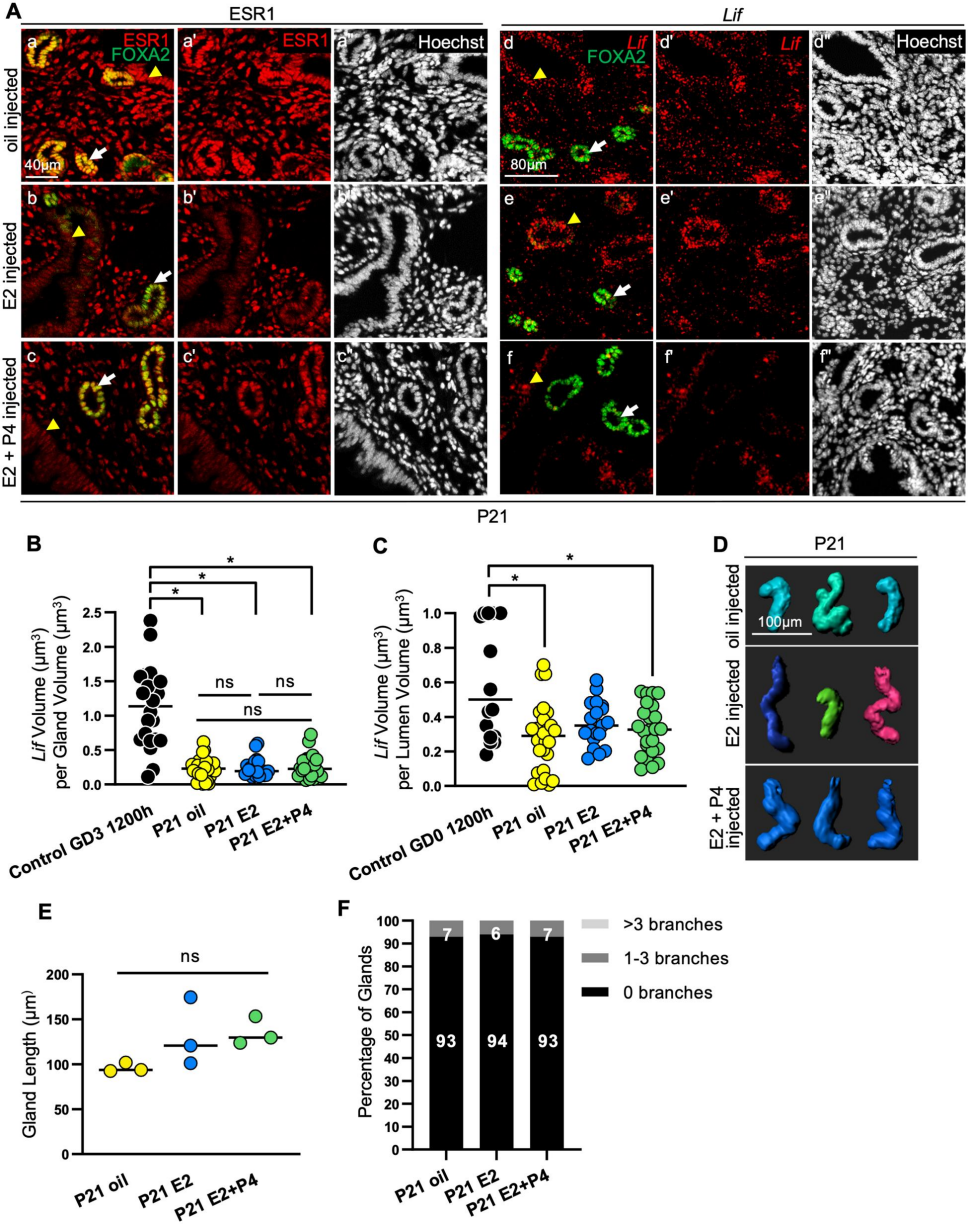
**Pre-pubertal, unbranched glands fail to produce leukemia inhibitory factor even when estrogen receptor 1 signaling is intact.** (**A**) Uterine sections of P21 control mice injected with either oil, E2, or E2 + P4 and stained for Hoechst (nuclei, white), FOXA2 (gland marker, green), ESR1 (a–c, red), and *Lif* mRNA (d–f, red). Scale bar, 40 μm (a–c). Scale bar, 80 μm (d–f). n = 9 regions each from three mice per treatment. Quantitative analysis of (**B**) the amount of *Lif* normalized for gland volume and (**C**) the amount of *Lif* normalized for lumen volume. n = 9 regions each from three mice per treatment. (B and C) Data analyzed using Mann–Whitney test. (*) = *P* *<* 0.05. Yellow arrowheads point towards the luminal epithelial cells and white arrows points towards the glandular epithelial cells (**D**) Representative 3D reconstructions of P21 control uterine glands with various treatments. n = 3 mice per treatment group. Scale bar, 100 μm. Quantitative analyses of (**E**) average gland length measurements (each dot represents one mouse) and (**F**) percentage of glands with 0, 1–3, and >3 branches in P21 control mice with various treatments. Hormonal treatment does not affect gland length or gland branching. 85–450 glands analyzed per mouse. (E) Data analyzed using Mann–Whitney test. (*) = *P <* 0.05. Two-proportion *Z*-test determined that the differences in percentage of gland branches between oil, E2, and E2 + P4 treatment is not statistically significant. FOXA2, forkhead box A2; ESR1, estrogen receptor 1; LIF, leukemia inhibitory factor; P, postnatal day; GD, gestational day; E2, estrogen; P4, progesterone.

XER glands are unbranched but also lack ESR1 signaling. To separate the contributions of gland branching and ESR1 signaling for glandular *Lif* expression, we used pre-pubertal P21 mice with unbranched glands and treated them with either E2 alone, E2 + P4, or vehicle (sesame oil) to evaluate the effect of ovarian steroids on *Lif* expression. We observed that P21 glandular epithelium, luminal epithelium and stroma express high levels of ESR1 in the presence of vehicle alone whereas ESR1 levels are reduced in the epithelium in the presence of two doses of E2 (Fig. 6A, a–a″, b–b″). On the other hand, ESR1 is highly expressed in glandular epithelium when P21 mice are treated with E2 and then P4 (Fig. 6A, c–c″). We also observed that P21 glands treated with vehicle or hormones express *Lif* in their luminal epithelium (Fig. 6A d–d″, e–e″, f–f″ and C). Finally, although we observed varying levels of ESR1 expression in the glandular epithelium of mice treated with vehicle or hormones, E2 treatment failed to elicit *Lif* expression in glandular epithelium (Fig. 6A and B). It has been suggested that glandular *Lif* expression occurs under P4 dominant conditions (Ma *et al.*, 2003); therefore, we also evaluated P21 mice treated with both E2 and P4 but again did not detect any *Lif* expression in the glands (Fig. 6A, f–f″). When we evaluated the 3D gland structure of the P21 uteri treated with different hormonal regimens (Fig. 6D), we observed that treatment with E2 alone or E2 + P4 did not result in a change in gland length compared to vehicle treatment (Fig. 6E). Further, in all treatment groups, glands were found to be primarily unbranched (Fig. 6F) and no glands with >3 branches were observed. Since ESR1 was expressed in the glandular compartment, the observed lack of *Lif* expression at P21 cannot be related to an absence of ESR1 signaling. Thus, branchless glands, even in the presence of E2–ESR1 signaling, fail to produce *Lif*.

## Discussion

Uterine glands are exocrine glands with a distinct structure that play vital roles in implantation and pregnancy success. Exocrine gland structure is well-known to contribute to gland function; however, such structure–function relationships for uterine glands have not been described. In our study, we discovered that developmental ESR1 signaling in different compartments of the murine uterus is necessary to build a branched gland. Further, we determine that both the branched structure of a uterine gland and ESR1 signaling are critical for gland function; specifically, preimplantation LIF production that is necessary for successful implantation.

### Secretory epithelial domains in branched uterine glands

During development, mouse uterine glands bud off the uterine lumen (Branham *et al.*, 1985) at P4. Glands elongate into the stroma, going through changes in morphology, including teardrop, elongated, and sinuous stages, until some begin branching at P21 (Vue *et al.*, 2018). In the adult mouse, uterine glands are prominently branched (Arora *et al.*, 2016). Human uterine glands in the functionalis and the basalis layer have also been characterized for 3D structure (Yamaguchi *et al.*, 2021; Tempest *et al.*, 2022). Glands in the functionalis layer are coiled and show some branching, while glands in the basalis layer show more prominent branching compared to the functionalis glands. Exocrine glands are generally classified as tubular or branched. Tubular exocrine glands do not have designated secretory cells, although tubular sweat glands have a coiled portion that holds secretions (Kurata *et al.*, 2017). In contrast to tubular glands, branched exocrine glands have acinar end-pieces that are secretory in nature (de Paula *et al.*, 2017). The presence of functional sub-structures in uterine glands (end-pieces, ducts, coils) has not been established. It is possible that the individual uterine gland has a ductal portion and a secretory end-piece (the branched end); however, it is also possible that individual uterine glands are end-pieces, and the uterine lumen accumulates the secretions as ducts do in other branched exocrine glands. Our study establishes that, in mice, unbranched uterine glands (*Pax2^Cre^*; *Esr1^flox/flox^*, and P21) are unable to produce *Lif.* This supports the idea that branched portions of uterine glands may carry the secretory function. Whether this is true for human uterine glands will be an avenue for future investigation.

### ESR1 signaling and gland structure in mammary and uterine glands

E2 signaling plays a key role in establishing the branching pattern of the exocrine mammary glands that are essential for lactation (Sternlicht *et al.*, 2006; Feng *et al.*, 2007). A whole-body deletion of *Esr1* leads to mammary glands with minimal branching (Bocchinfuso *et al.*, 2000; Sternlicht *et al.*, 2006), while a mammary epithelium-specific deletion of *Esr1* displays a loss of duct elongation and side branching (Feng *et al.*, 2007). Thus, gland proliferation and branching are ESR1 signaling-dependent in the mammary gland. In mice with continuous estrous, the uterine glands display a cribriform structure (Stewart *et al.*, 2022), suggesting that aberrant ESR1 signaling must impact uterine gland shape. In our study, we determined that neonatal deletion of ESR1 in the epithelial and stromal compartments resulted in gland buds, while deletion only in the epithelial compartment resulted in glands that elongate but fail to branch. Together, this suggests that stromal ESR1 signaling regulates gland bud outgrowth and elongation in a non-cell-autonomous manner while epithelial ESR1 signaling regulates uterine gland branching in a cell-autonomous manner. In the mammary gland, E2–ESR1 signaling regulates bud growth and branching morphogenesis in conjunction with Fgf10, Wnt, and heparan sulfate proteoglycan signaling (Khan *et al.*, 2022). Signaling pathways interacting with E2–ESR1 signaling that are critical for bud elongation and branching still remain to be discovered and will be a subject of future investigations.

The gland bud phenotype in *Pgr^Cre^*  *Esr1^flox/flox^* glands is remarkably similar to gland structure observed in uteri with neonatal deletion of *Foxa2* using the same *Pgr^Cre^* driver line (Fig. 4F in Matsuo *et al.* (2022)). Depletion of ESR1 in the adult epithelium using the *Ltf^Cre^* driver in our study did not cause any quantifiable structural gland phenotypes, suggesting that epithelial ESR1 signaling during puberty is required for gland branching. Once glands are branched, reduced E2–ESR1 signaling did not affect gland structure during estrous cycles. This is in contrast with loss of FOXA2 in the adult epithelium using the same *Ltf^Cre^* driver line, which results in loss of *Lif* and the gland structure appears abnormal (Fig. 4F in Matsuo *et al.* (2022)). We propose that ESR1 signaling and FOXA2 may work together for gland bud elongation and for *Lif* production; however, they may be redundant to maintain branched glands post-puberty.

### Utility of *Pax2* Cre for studying uterine epithelium

Both *Wnt7a* and *Pax2* are expressed in the embryonic precursor to the uterus, the Müllerian duct (Douglas *et al.*, 2012). Thus, Cre lines driven by the promoters of these genes are useful tools to excise genes in the uterine epithelial cells. *Wnt7a^Cre^ ESR1^flox/flox^* mice do not support embryo entry into the uterus as the embryos die in the oviduct, precluding analysis of embryo implantation studies. Using the *Pax2^Cre^* mouse line, we were able to bypass the oviductal phenotype and embryos were observed in the uterus during the pre-implantation and implantation time points. This may be because we have incomplete and patchy expression of the *Pax2^Cre^* lineage in the oviductal epithelium. Recently, Hancock *et al.* (2023) determined that *Wnt7a^Cre^ ESR1^flox/flox^* mice also display ectopic expression of gland marker FOXA2 in the uterine luminal epithelium at GD3 1200 h. In *Wnt7a^Cre^* mouse, *Wnt7a* coding exons are replaced by *Cre*, making this model heterozygous for the *Wnt7a* gene (Winuthayanon *et al.*, 2010). It is unknown if the ectopic expression of FOXA2 in the *Wnt7a^Cre^ ESR1^flox/flox^* lumen is related to the depletion of ESR1 alone or to the additional loss of one copy of *Wnt7a*. WNT7A has key functions in uterine epithelium and gland specification (Dunlap *et al.*, 2011; Goad *et al.*, 2017) and could interfere with the ESR1 mutant analysis. We did not observe ectopic expression of FOXA2 in our *Pax2^Cre^ ESR1^flox/flox^* uterine lumen, and we observed severe gland branching phenotypes in both models where ESR1 signaling is disrupted in the embryonic epithelium. This supports the idea that alternate Cre lines, such as *Pax2^Cre^*, may be powerful tools to study the impact of loss of gene function in the embryonic uterine epithelium where *Wnt7a^Cre^* cannot be used.

### E2–ESR1 signaling and *Lif* production

LIF is an essential gland secretion that is absolutely necessary for implantation in mice. *Lif* knockout mice and other genetic models that fail to produce LIF exhibit embryo implantation failure. ESR1 signaling is predicted to be upstream of *Lif* expression because ESR1 binding sites are observed in the coding region and in the 3′ untranslated region of the *Lif* gene (Hewitt *et al.*, 2012). In uterine biology literature, often a bolus of E2 injection results in increased levels of *Lif* mRNA, which is usually observed using whole tissue analysis such as quantitative PCR. Since E2–ESR1 signaling can upregulate *Lif* in both luminal and glandular epithelium but only glandular LIF promotes embryo implantation, it is vital that these studies be interpreted carefully and the location of *Lif* upregulation be determined when employing E2 treatment. We observed that ovulatory E2 only induces high *Lif* expression in the luminal epithelium, while in the P4-dominant pre-implantation stages, *Lif* is confined to the glandular epithelium. It has been shown in various mammalian species, such as mice (Ma *et al.*, 2003), hamsters (Ding *et al.*, 2008), marmosets (Kholkute *et al.*, 2000), and rabbits (Yang *et al.*, 1996), that E2 alone is unable to induce *Lif* expression in the glands and other factors may be necessary for LIF production. Our work suggests that, in addition to E2–ESR1 signaling, the branched structure of the uterine gland is necessary for *Lif* synthesis. *Lif* mRNA was negligible when uterine glands lacked ESR1 signaling in addition to a branched structure. Similarly, intact E2–ESR1 signaling in unbranched prepubertal glands also resulted in low *Lif* mRNA. Our quantitative data from both mouse models with epithelial deletion of ESR1 suggests that glands with greater than three branches are key for producing threshold levels of glandular LIF to support implantation resulting in pregnancy success. We also observed that uteri with normally branched glands but severely reduced ESR1 signaling were still able to produce threshold levels of LIF to support embryo implantation and pregnancy success. This indicates that gland functional ability (LIF secretion and implantation) is dependent on its structure, particularly whether a gland is branched or unbranched.

### Uterine gland structure–function relationships

Loss of ESR1 signaling in the mammary glands results in a poorly developed branched structure and compromises the lactation function of the glands. We also show that unbranched glands compromise *Lif* secretion, resulting in implantation failure, and this phenotype is partially rescued when pregnancy is supplemented with LIF. It is critical to note that supplemental LIF can rescue both implantation and pregnancy, at least partially, in genetic mutants where glands are branched but simply fail to produce LIF (adult epithelial ESR1 deletion, this study, and adult epithelial FOXA2 deletion (Kelleher *et al.*, 2017)). However, if a genetic mutant displays failure of glands to branch and fails to produce *Lif*, then implantation is rescued (embryonic ESR1 deletion, this study and neonatal FOXA2 deletion (Dhakal *et al.*, 2021; Kelleher *et al.*, 2017; Matsuo *et al.*, 2022)) but pregnancy fails to continue post-implantation (Kelleher *et al.*, 2017). These data support essential functions for branched uterine glands in the post-implantation phase. Reduced levels of LIF are present in the uterine luminal fluid of infertile women (Lass *et al.*, 2001; Tawfeek *et al.*, 2012); however, LIF is unable to rescue implantation in case of assisted reproduction in women with recurrent unexplained implantation failure (Brinsden *et al.*, 2009; Aghajanova, 2010). Nevertheless, in addition to LIF, gland structure (branching and coiling) may be key to glandular secretions that may be critical for human implantation.

Mice with neonatal or adult epithelial deletion of FOXA2 displayed implantation failure, but the embryos apparently entered a diapause state. Embryos in our embryonic epithelial deletion of ESR1 did not survive beyond implantation stages. This could be linked to differences in the progesterone signaling in these mutant mice and will be a subject of future studies. However, even with embryos that entered diapause in FOXA2 deletion mutants, the viability of embryos that failed to implant was higher if glands were branched compared to bud-staged glands (Matsuo *et al.*, 2022), supporting multiple roles for branched uterine glands beyond LIF secretion and embryo implantation.

From our research into the structure–function relationship of uterine glands in the context of pregnancy outcomes, we have gathered new insights regarding how uterine estrogen signaling affects gland development and structure, and how gland structure contributes to the induction of *Lif* and events surrounding embryo implantation. To expand the knowledge of how branched and unbranched glands differ in functional capability, further research is needed to identify the secretory factors a branched gland makes and if there are specific secretory cell types in uterine glands, similar to mammary and salivary glands (Khan *et al.*, 2022). Additionally, it will be useful to assess the cellular differentiation events that define how glands bud off the main luminal epithelial tube, the steroid hormone-dependent and independent signaling mechanisms, and the changes in the extracellular matrix that guide gland bud elongation and branching. Further, post-pubertal gland development, especially in the context of regeneration in the post menses and the post-partum uterus, remain understudied. More research is therefore needed to characterize how cyclicity and pregnancy remodel uterine glands as well as how new gland buds may arise in adulthood. Gland structure–function dynamics in humans are challenging as it will be necessary to assess both the basalis and the functionalis layers of endometrial glands. Therefore, the mouse can provide novel insights into gland structure–function relationships relevant to early pregnancy in mammals.

## Supplementary Material

gaae020_Supplementary_Data

## Data Availability

Data generated in the article is available upon reasonable request to the corresponding author.

## Author Biography

**Dr. Ripla Arora:** Dr. Arora is an Associate professor in the Department of Obstetrics, Gynecology, and Reproductive Biology and the Institute for Quantitative Health Science and Engineering at Michigan State University. Dr. Arora received her PhD degree in Genetics and Development from Columbia University where she studied the role of T-box transcription factors in umbilical vessel and lung development. As a post-doctoral fellow at the University of California, San Francisco, she developed a novel uterine 3D imaging and quantitative modeling method. Her research focuses on uncovering 3D structure-based mechanisms that guide both development of the uterus and early embryo–uterine interactions for implantation success.
