# Supplementary material for: Murine uterine gland branching is necessary for gland function in implantation
Source: Mol Hum Reprod. 2024 May 24;30(6):gaae020. doi: 10.1093/molehr/gaae020 (PMC11176042; doi:10.1093/molehr/gaae020)
Supplement: gaae020_Supplementary_Data [file gaae020_supplementary_data.zip › Granger et al Supplementary Information 5_14_24.pdf]

Murine uterine gland branching is necessary for gland function in implantation

Katrina Granger[1,2,†], Sarah Fitch[1,2,†], May Shen[1,2], Jarrett Lloyd[1,2], Aishwarya Bhurke[1,2], Jonathan Hancock[3,4], Xiaoqin Ye[3,4], Ripla Arora[1,2,*]

Supplementary Figure S1. *Pax2 lineage in oviductal epithelium*
Supplementary Figure S2. *ESR1 is not expressed in uterine vasculature.*
Supplementary Figure S3. *3D reconstructions of uterine lumen and glands.*
Supplementary Figure S4. *Neonatal and embryonic deletion results in reduced gland length during implantation.*
Supplementary Figure S5. *Gland branching is disrupted when two different Cre lines are used for embryonic epithelial Esr1 deletion.*
Supplementary Figure S6. *Differential Lif expression in luminal and glandular epithelium in early pregnancy.*
Supplementary Figure S7. *Implantation rescue in ESR-1 depleted mice with supplemental Lif.*

Supplementary Table S1. *Statistical analysis of gland branch numbers in control and XER mice at various stages.*

Supplementary Figure S1

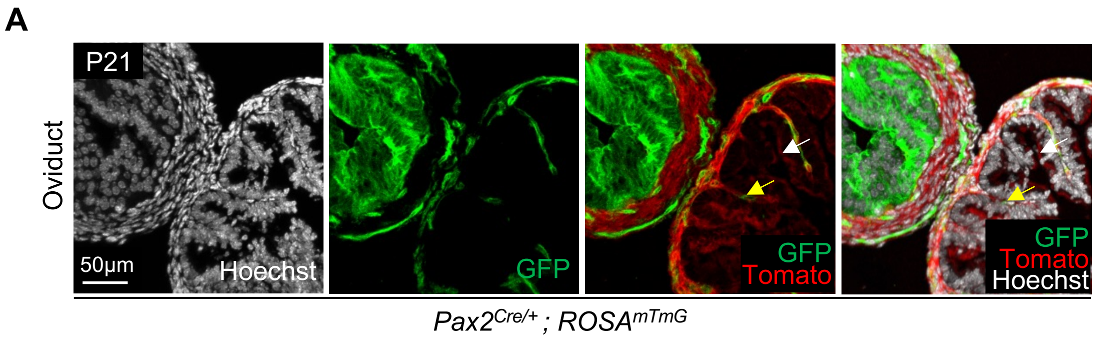

Supplementary Figure S1. *Pax2 lineage in oviductal epithelium* (A) *Pax2*$^{Cre/+}$ *; ROSA*$^{mT/mG}$ mouse oviduct with Hoechst (nuclei, white), GFP (green), and Tomato (red) demonstrating patchy Pax2 lineage GFP reporter expression in the oviductal epithelium. Scale bar, 50µm. Yellow arrows indicate GFP lineage expression in oviductal epithelium. White arrows indicate Tomato expression and absence of GFP expression in oviductal epithelium. Abbreviations: (P) postnatal day.

Supplementary Figure S2

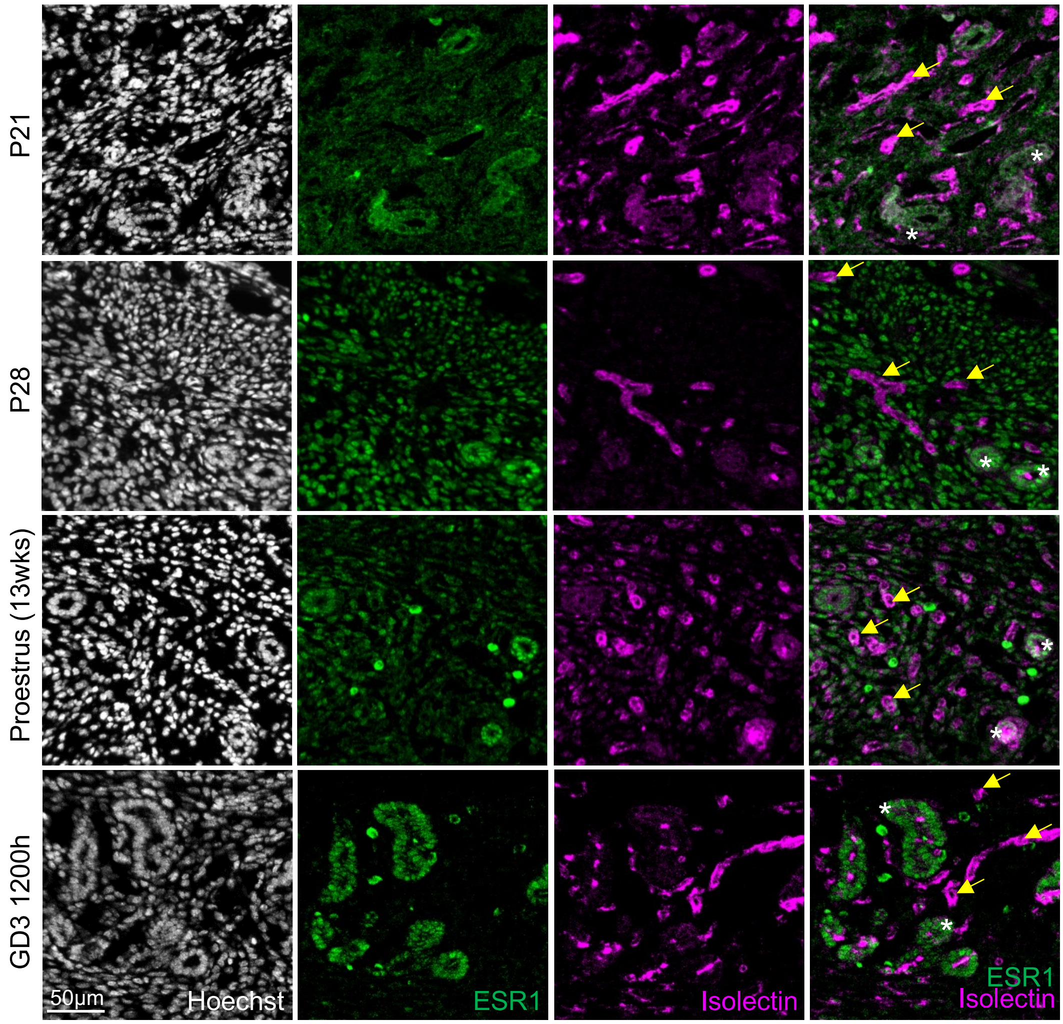

Supplementary Figure S2. *ESR1 is not expressed in uterine vasculature.* Uterine sections from control mice stained with Hoechst (nuclei, white), ESR1 (green), and Isolectin (endothelial marker, magenta). Scale bar, 50µm. P21, n=2 mice; P28, Proestrus and GD3 1200h n=3 mice per age/stage. Yellow arrows indicate regions of vasculature and white asterisks indicate cells expressing ESR1. No overlap is observed between the two. Abbreviations: (P) postnatal day, (GD) gestational day.

Supplementary Figure S3

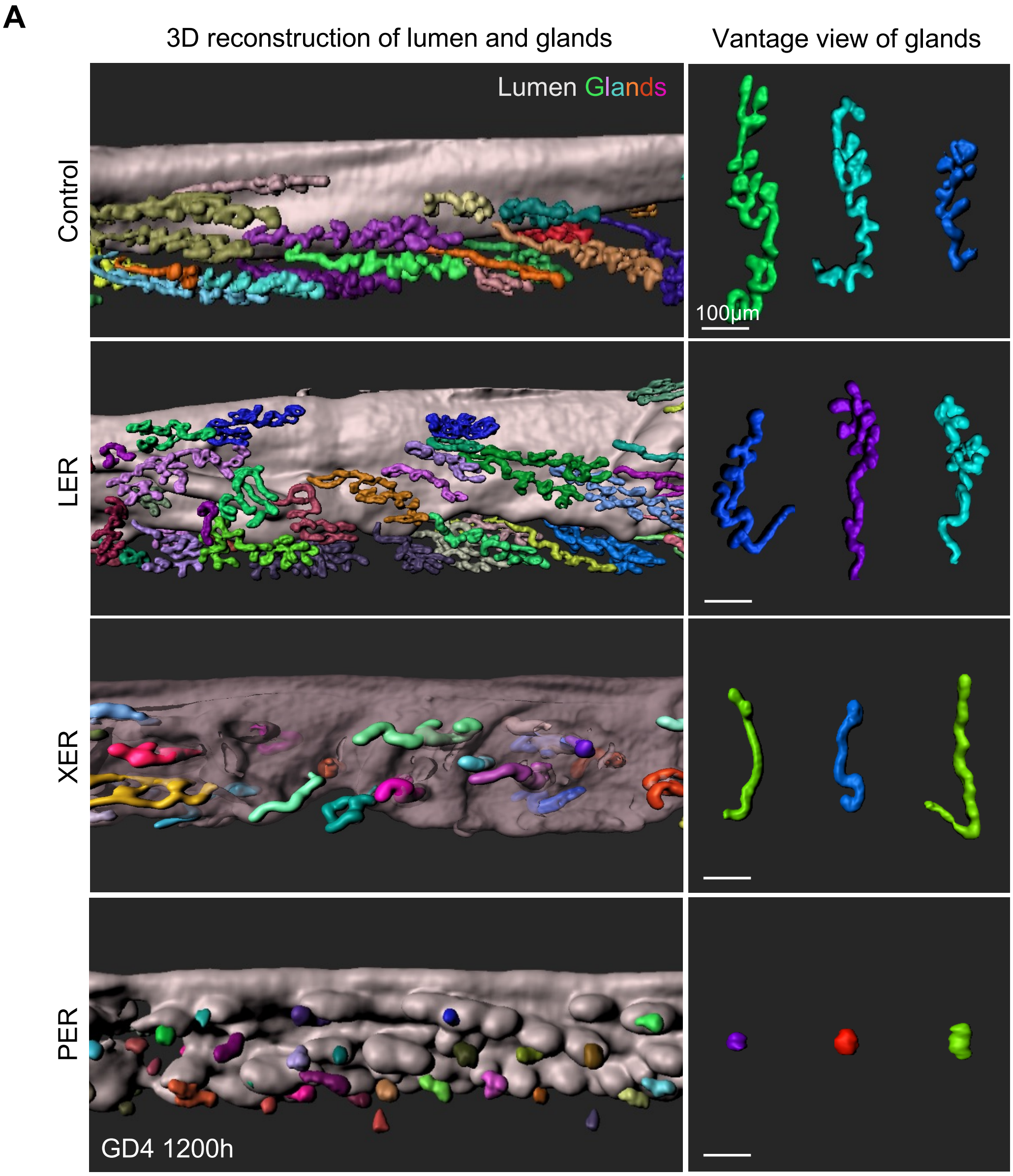

Supplementary Figure S3. *3D reconstructions of uterine lumen and glands.* (A) Whole uterine tissue from GD4 1200h control, LER, XER, and PER mice stained with CDH1 (epithelial marker) and FOXA2 (gland marker). Following confocal imaging at 10x magnification, 3D surfaces (left) of lumen and glands were made for visualization of structures. Vantage view (right) of glands allows for comparison of individual glands. Scale bar, 100µm. Ages of control, XER, and PER mice are 6-8 weeks, and ages of LER mice are 12-14 weeks. Abbreviations: (GD) gestational day, (LER) *Ltf^{Cre} $ESR1^{flox/flox}$*, (XER) *$Pax2^{Cre}$ $ESR1^{flox/flox}$*, (PER) *Pgr^{Cre} $ESR1^{flox/flox}$*.

Supplementary Figure S4

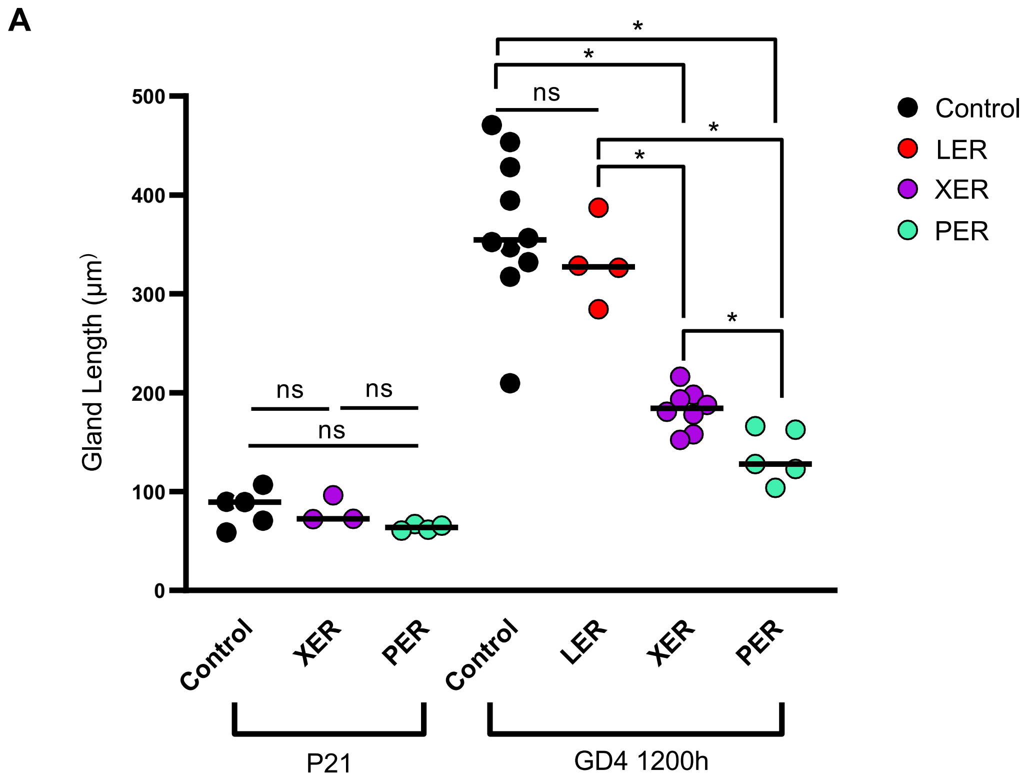

Supplementary Figure S4. *Neonatal and embryonic deletion results in reduced gland length during implantation.* (A) Quantitative analysis of average gland length measurements at P21 and GD4 1200h. (n=3) mice/genotype/stage. 75-400 glands analyzed per mouse. Dot represents average gland length measurement from a single mouse. Data analyzed using Mann-Whitney test (*) = $p<0.05$. (ns) = $p>0.05$. Abbreviations: (P) postnatal day, (GD) gestational day, (LER) Ltf^{Cre} $ESR1^{flox/flox}$, (XER) $Pax2^{Cre}$ $ESR1^{flox/flox}$, (PER) Pgr^{Cre} $ESR1^{flox/flox}$.

Supplementary Figure S5

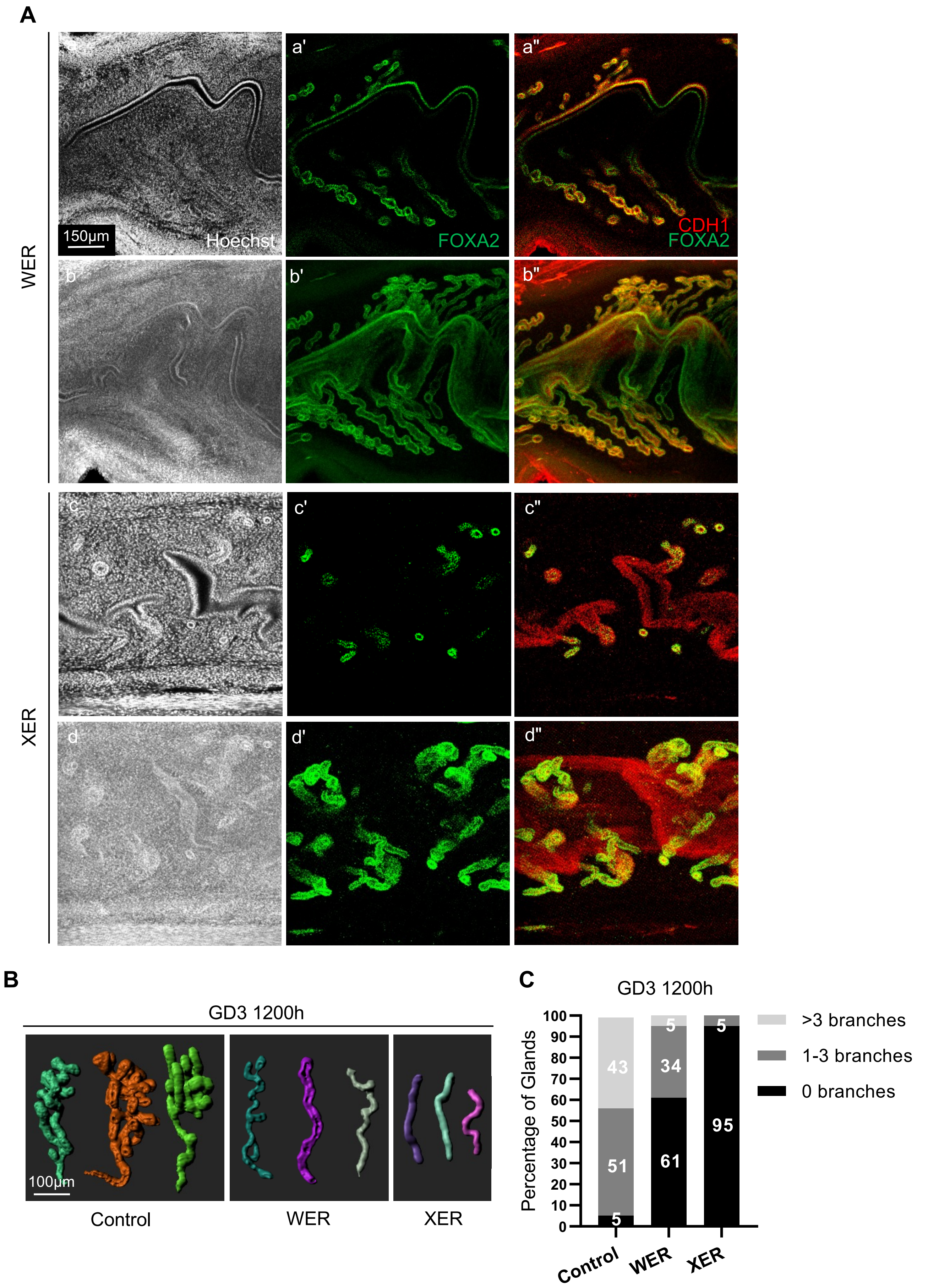

Supplementary Figure S5

Supplementary Figure S5. *Gland branching is disrupted when two different Cre lines are used for embryonic epithelial Esr1 deletion.* (A) Immunofluorescent images of (a-b) WER and (c-d) XER GD3 1200h uterine lumen and glands stained with Hoechst (nuclei, white), FOXA2 (gland marker, green), and CDH1 (epithelium, red). (a, a′, a″, c, c′, c″) Indicates 7μm slice view. (b, b′, b″, d, d′, d″) Indicates pseudo-3D view (150μm slice). Scale bar, 150μm. (B) Representative 3D reconstructions of control, WER, and XER glands previously stained with gland marker FOXA2 and imaged using a confocal microscope. Scale bar, 100μm. (n=3) mice. (C) Quantitative analysis of percentage of glands with 0, 1-3, and >3 branches in control, WER, and XER mice at GD3 1200h. Controls (n=2 mice, 2 uterine horns); WERs and XERs (n=2 mice, 4 uterine horns). 100-375 glands analyzed per mouse. Two-proportion Z-test determined that the differences in percentage of gland branches between control and WER glands, control and XER glands and WER and XER glands is statistically significant. Abbreviations: (GD) gestational day, (WER) *Wnt7a^{Cre} ESR1$^{flox/flox}$*, (XER) *Pax2^{Cre} ESR1$^{flox/flox}$*.

Supplementary Figure S6

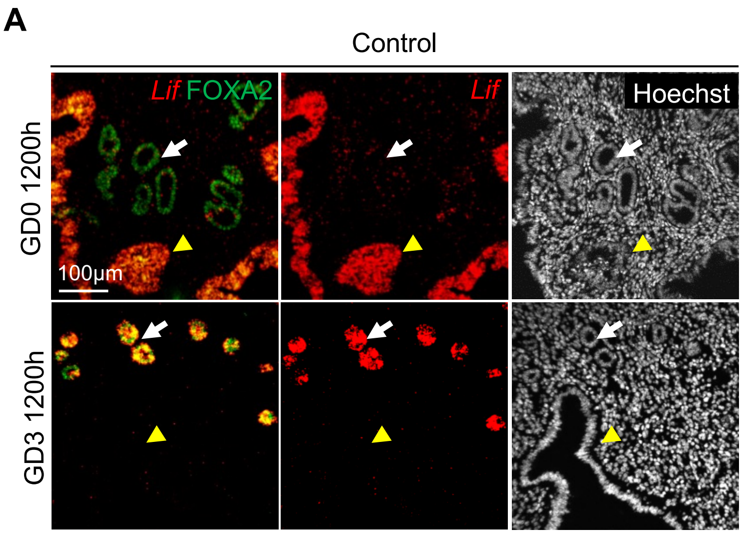

Supplementary Figure S6. *Differential Lif expression in luminal and glandular epithelium in early pregnancy.* (A) Uterine section of control mouse at GD0 1200h and GD3 1200h with staining for Hoechst (nuclei, white), FOXA2 (gland marker, green), and *Lif* mRNA (red). *Lif* expression is evident in only the luminal epithelium at GD0 1200h and only the glandular epithelium at GD3 1200h. White arrows indicate glandular epithelium and yellow arrowheads indicate luminal epithelium. Scale bar, 100µm. (n=3) mice per stage. Abbreviations: (GD) gestational day.

Supplementary Figure S7

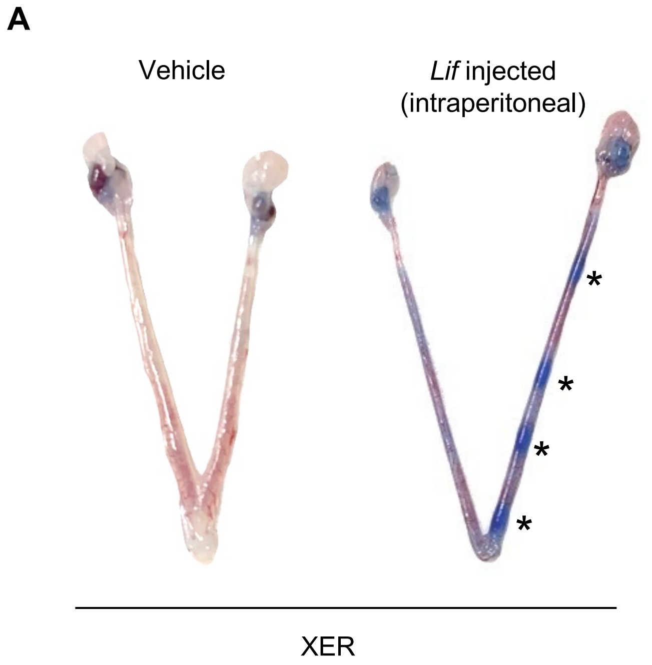

Supplementary Figure S7. *Implantation rescue in ESR-1 depleted mice with supplemental Lif.* (A) Dissected GD4 1200h uteri from XER mice injected intraperitoneally with either 10µg *Lif* or vehicle (PBS). Asterisks indicate blue dye sites. Abbreviations: (XER) *Pax2*^{Cre} *ESR1*$^{flox/flox}$.

Supplementary Table S1. *Statistical analysis of gland branch numbers in control and XER mice at various stages. Two Proportion Z-test performed*

0 branches	Control P21	Control P28	Control P35	Control P42 GD4 1200h	Control >11wk GD4 1200h	XER P21	XER P28	XER P35	XER P42 GD4 1200h
Control P21	-	-	-	-	-	-	-	-	-
Control P28	S	-	-	-	-	-	-	-	-
Control P35	-	S		-	-			-	-
Control P42 GD4 1200h	-	-	S	-	-	-	-	-	-
Control >11wk GD4 1200h	-	-	-	S	-	-	-	-	-
XER P21	S	-	-	-	-	-	-	-	-
XER P28	-	NS	-	-	-	S	-	-	-
XER P35	-	-	S	-	-	-	NS	-	-
XER P42 GD4 1200h		-	-	S	-	-	-	$p<0.03$	-

S: significant, $p<0.05$

NS: not significant

1-3 branches	Control P21	Control P28	Control P35	Control P42 GD4 1200h	Control >11wk GD4 1200h	XER P21	XER P28	XER P35	XER P42 GD4 1200h
Control P21	-	-	-	-	-	-	-	-	-
Control P28	S	-	-	-	-	-	-	-	-
Control P35	-	S		-	-			-	-
Control P45 GD4 1200h	-	-	S	-	-	-	-	-	-
Control >11wk GD4 1200h	-	-	-	S	-	-	-	-	-
XER P21	S	-	-	-	-	-	-	-	-
XER P28	-	NS	-	-	-	S	-	-	-
XER P35	-	-	S	-	-	-	NS	-	-
XER P42 GD4 1200h		-	-	S	-	-	-	$p<0.03$	-

S: significant, $p<0.05$

NS: not significant

>3 branches	Control P21	Control P28	Control P35	Control P42 GD4 1200h	Control >11wk GD4 1200h	XER P21	XER P28	XER P35	XER P42 GD4 1200h
Control P21	-	-	-	-	-	-	-	-	-
Control P28	NS	-	-	-	-	-	-	-	-
Control P35	-	NS		-	-			-	-
Control P45 GD4 1200h	-	-	S	-	-	-	-	-	-
Control >11wk GD4 1200h	-	-	-	S	-	-	-	-	-
XER P21	NS	-	-	-	-	-	-	-	-
XER P28	-	NS	-	-	-	NS	-	-	-
XER P35	-	-	NS	-	-	-	NS	-	-
XER P42 GD4 1200h		-	-	S	-	-	-	NS	-

S: significant, $p<0.05$

NS: not significant

Abbreviations: (P) postnatal day, (GD) gestational day, (XER) *$Pax2^{Cre}$ $ESR1^{flox/flox}$*
