# Supplementary figures and images for: Murine uterine gland branching is necessary for gland function in implantation
Source: Mol Hum Reprod. 2024 May 24;30(6):gaae020. doi: 10.1093/molehr/gaae020 (PMC11176042; doi:10.1093/molehr/gaae020)

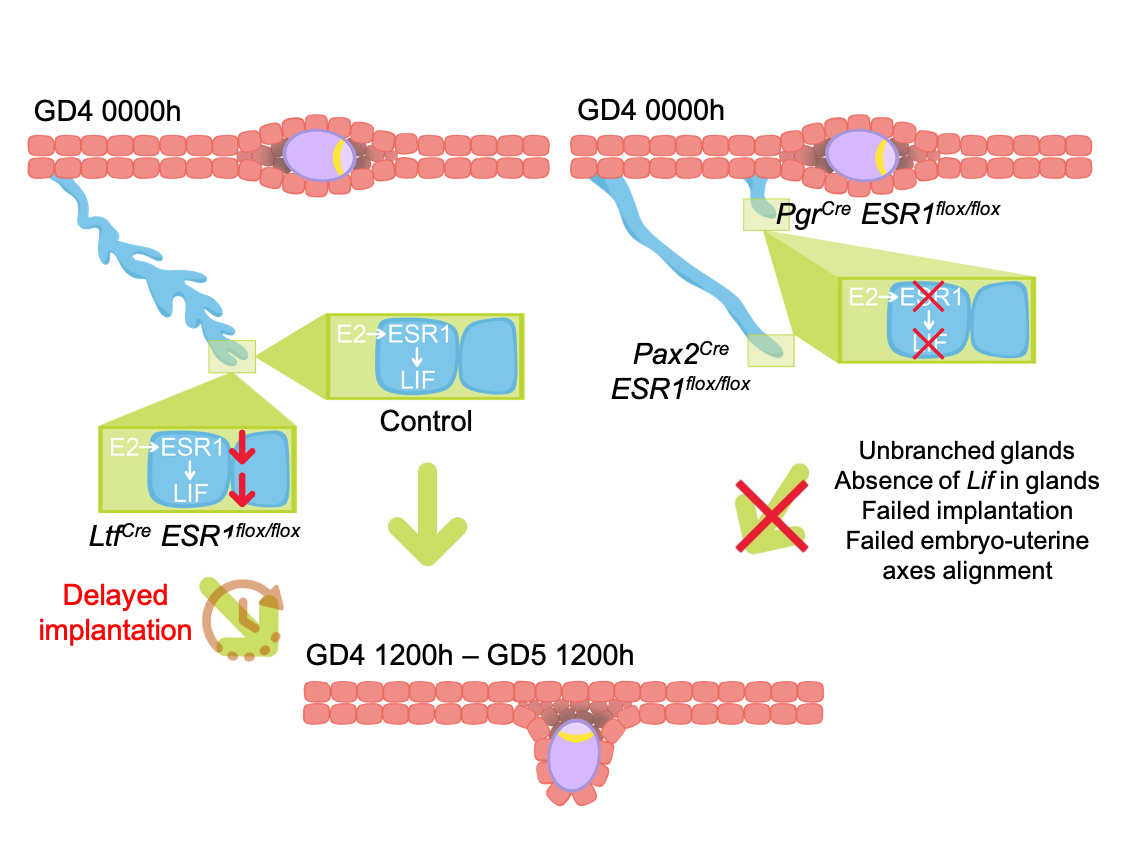

Supplement: gaae020_Supplementary_Data [file gaae020_supplementary_data.zip › Graphical Abstract.png]
